# 2-octyl cyanoacrylate sealing of the pancreatic remnant after distal pancreatectomy – A prospective pilot study

**DOI:** 10.1371/journal.pone.0205748

**Published:** 2018-10-16

**Authors:** Felix J. Hüttner, Pascal Probst, Hannes G. Kenngott, Phillip Knebel, Thilo Hackert, Alexis Ulrich, Markus W. Büchler, Markus K. Diener

**Affiliations:** 1 Department of General-, Visceral- and Transplantation Surgery, University of Heidelberg, Heidelberg, Germany; 2 Study Center of the German Surgical Society, University of Heidelberg, Heidelberg, Germany; University of Cambridge, UNITED KINGDOM

## Abstract

**Background:**

Postoperative pancreatic fistula (POPF) remains a frequent problem especially after distal pancreatectomy. The application of 2-octyl cyanoacrylate showed promising results in the reduction of POPF after pancreatoduodenectomy prompting an expansion of this technique to distal pancreatectomy. Thus, the objective of the current study was to assess safety, feasibility and preliminary efficacy of an intraoperative 2-octyl cyanoacrylate application after distal pancreatectomy.

**Methods:**

Between April 2015 and June 2016 adult patients scheduled for elective distal pancreatectomy were considered eligible for the study. It was planned to include a total of 35 patients. After distal pancreatectomy with hand-sewn closure of the pancreatic remnant, a 2-octyl cyanoacrylate surgical glue was applied to the cut surface of the pancreas. Patients were followed up for three months with main focus on safety in terms of (serious) adverse events. Further endpoints included POPF, other pancreas-specific and surgical complications.

**Results:**

15 patients were included in the study because the manufacturer stopped production and distribution of the investigational device thereafter. There was a total of ten serious adverse events but no device-related events and no mortality. The serious adverse events depicted a typical safety profile after distal pancreatectomy. POPF occurred in five cases (33.3%), delayed gastric emptying and post-pancreatectomy haemorrhage in two cases respectively (13.3%).

**Conclusions:**

Application of 2-octyl cyanoacrylate to the pancreatic remnant after distal pancreatectomy seems feasible and safe. The planned evaluation of preliminary efficacy was not possible due to the inadvertent early termination and subsequent small sample size of the study. Novel techniques for prevention and therapy of POPF should be evaluated in future trials.

## Introduction

Pancreatic surgery is complex from a diagnostic, surgical and perioperative point of view. Centralization of pancreatic surgery in specialized institutions has led to acceptable mortality rates below 5%. [1–3] Moreover, standardization of surgical and perioperative care in these centers of expertise is a prerequisite for low morbidity rates. [2]

However, postoperative pancreatic fistula (POPF) still represents the most common postoperative morbidity in pancreatic surgery and can profoundly affect patient recovery and outcome. Especially after distal pancreatectomy, the rates remain at a high level of > 30% in various recent trials. [4–6] In-hospital mortality due to POPF or subsequent complications occurs in up to 14%, [7] reaching up to 33% in high-risk subgroups. [8] Several surgical techniques and technical modifications have been proposed in an attempt to reduce fistula rates in pancreatic surgery. [9] For instance, different types of fibrin sealants have been evaluated in their potential to reduce the occurrence of POPF, but none of them has been proven effective so far. [10, 11] Also, mesh reinforcements of the pancreaticojejunal anastomosis have provided no significant benefit in the reduction of POPF. [12] Currently, both scalpel transection with hand-sewn closure as well as stapler closure of the pancreatic remnant can be regarded as reference standards in distal pancreatectomy. [13]

Compared to fibrin sealants, the medical glue 2-octyl cyanoacrylate (2-OCA) is also easily applicable to the resection surface and is not degraded by aggressive pancreatic enzymes due to its long-lasting tissue bonds. Cyanoacrylate is an acrylic resin that rapidly polymerizes in the presence of water, forming, long, strong bonds that join surfaces together. The compound 2-OCA is a nontoxic bacteriostatic medical glue that has been widely used to approximate skin edges. [14]

In 2013, Barakat et al. [15] have published their first results on the topical application of 2-OCA to the pancreaticojejunal anastomosis after pancreatoduodenectomy. They reported a highly significant reduction of POPF for the 2-OCA group compared to patients without 2-OCA application (POPF rate: 3.5% vs. 36%).

Based on the results of Barakat et al. [15], topical 2-OCA application promised a substantial benefit in the prevention of POPF. Therefore, an expansion of this promising technique to distal pancreatectomy, which shows even higher rates of POPF compared to pancreatoduodenectomy, seemed reasonable. The objective of the current pilot study was to assess safety and feasibility and to create preliminary efficacy data forming the basis for a subsequent randomized controlled trial.

## Methods

The study was conducted as a prospective, monocenter proof-of-concept study at the development stage according to the IDEAL recommendations [16] with the main objective to evaluate safety and feasibility of the intervention; therefore no actual sample size calculation was performed. It was planned to include a total of 35 patients, which was judged sufficient by a board of trial experts for a preliminary evaluation of safety and applicability in this early development phase of the new technique. [17] Regarding preliminary efficacy, the POPF rate after distal pancreatectomy without additional sealing was about 30% in previous prospective trials [4, 18] and based on the results by Barakat et al. [15] a substantial reduction of POPF of approximately 10–15% by the study intervention was expected. Therefore, a number of 30 evaluable patients, taking into account potential drop-out of maximally 5 patients, was considered sufficient to reveal a preliminary difference for formal sample size calculation of a subsequent larger randomized controlled trial. The study was conducted at the Department of General, Visceral, and Transplantation Surgery of the University of Heidelberg, Germany. The study was prospectively registered in the German Clinical Trials Register DRKS00007915 on April 9^th^ 2015.

The study was performed under the regulations of the German Medical Devices Act and in conformance with the Declaration of Helsinki and ICH GCP. The study was approved by the Independent Ethics Committee of the University of Heidelberg (approval reference number: MZmo-577/2014) and by the competent German authority (Federal Insititute for Drugs and Medical Devices, BfArM; approval reference number: 94.1.07–5660–9362) before inclusion of the first patient. All patients provided written informed consent after comprehensive information by an investigator of the study before any study-specific procedures took place.

Adult patients scheduled for elective distal pancreatectomy due to any underlying disease were considered eligible for the study. Exclusion criteria are listed in Table 1.

**Table 1:** Exclusion criteria.

Haemoglobin< 10 g/dl	Immunosuppressive therapy (cortison ≥ 40 mg/d or equivalent; azathioprin)
Bilirubin > 3 times ULN	Pregnancy or lactation
AST or ALT > 4 ULN	Drug trial participation within 30 days before screening visit
INR > 1.7	Understanding or language problems
Creatinine clearance < 30 ml/min (estimated by Cockcroft-Gault)	Inability to comply with study and/or follow-up procedures
Serious cardiovascular disease (e.g. myocardial infarction in the last 12 months, congestive heart failure NYHA III/IV, unstable angina pectoris)	Allergy or known intolerability to 2-OCA, butyl-lactoyl cyanoacrylate or formaldehyde
Liver cirrhosis (of any Child-Pugh grade)	Any condition which could result in an undue risk for the patient in the opinion of the investigator
ASA score > III	

The investigational device was the commercially available CE marked and certified surgical sealant OMNEX, manufactured for ETHICON by Closure Medical Corp., Raleigh, North Carolina, 27616. OMNEX is a synthetic tissue sealant consisting of a blend of two monomers, 2-OCA and butyl-lactoyl-cyanoacrylate, which creates a flexible physical seal after polymerization, independent of the body’s clotting mechanism. During a period of approximately 36 months, the sealant eventually degrades via hydrolytic chain scission, breaking down into smaller absorbable fragments.

After routine resection of the pancreatic tail and/or body, the remnant was closed according to local standards [18] by direct suture of the pancreatic duct with a non-absorbable surgical suture (e.g. Novafil 4–0) and closure of the pancreatic cut surface with absorbable sutures (e.g. PDS 5–0) in fish-mouth technique. Afterwards, a thin layer of the 2-OCA surgical sealant was applied to the sutured surface of the pancreatic remnantt. The surrounding area was covered with sterile surgical gauzes to avoid contact of the sealant to other tissue not intended to get in contact with the sealant. Before application, the surface of the pancreatic remnant was patted dry with a sterile gauze, to assure direct contact of the sealant to the tissue as described in the directions for use of the product. No additional covering of the pancreatic remnant such as teres ligament patch or similar procedures were performed to avoid confounding. After polymerization of at least 2–3 minutes the operation was continued in a routine manner. [18]

Patients treated with the study intervention were followed-up for a total of 3 months. The main outcome parameter was safety assessed by the frequency of serious adverse events and device-related adverse events. Further endpoints included the following surgical complications: 30-day mortality, occurrence of postoperative pancreatic fistula within 30 days after surgery according to the ISGPF definition, [19] delayed gastric emptying, postpancreatectomy hemorrhage both according to the respective ISGPS definition, [20, 21] postoperative pancreatitis, intra-abdominal abscess or fluid collection, relaparotomy, burst abdomen, wound infection. In addition, medical complications such as perioperative myocardial infarction, perioperative cerebral vascular incidents, perioperative deep vein thrombosis and pulmonary embolism were assessed. As a measure of overall morbidity, the comprehensive complication index was assessed. [22] Finally, duration of surgery, intraoperative blood loss and length of hospital stay were evaluated.

All patients treated with the study intervention were considered in the final analysis. The empirical distribution of all baseline characteristics and endpoints was calculated, including median, range and quartiles in case of continuous variables and scores, and with absolute and relative frequencies in case of categorical data. 95% confidence intervals were calculated.

(Serious) adverse events were summarized using descriptive statistics. The adverse events were categorized as surgical, cardiovascular, pulmonary, urinary and other complications. Furthermore, it was assessed if events were device-related or not. Device-related and not device-related (serious) adverse events were reported separately. Proportions and frequencies of adverse events were presented with specific focus on potential device-related adverse events. All statistical analyses were conducted with R statistical software (R Foundation for Statistical Computing, Vienna, Austria. http://www.R-project.org).

## Results

**Fig 1:**
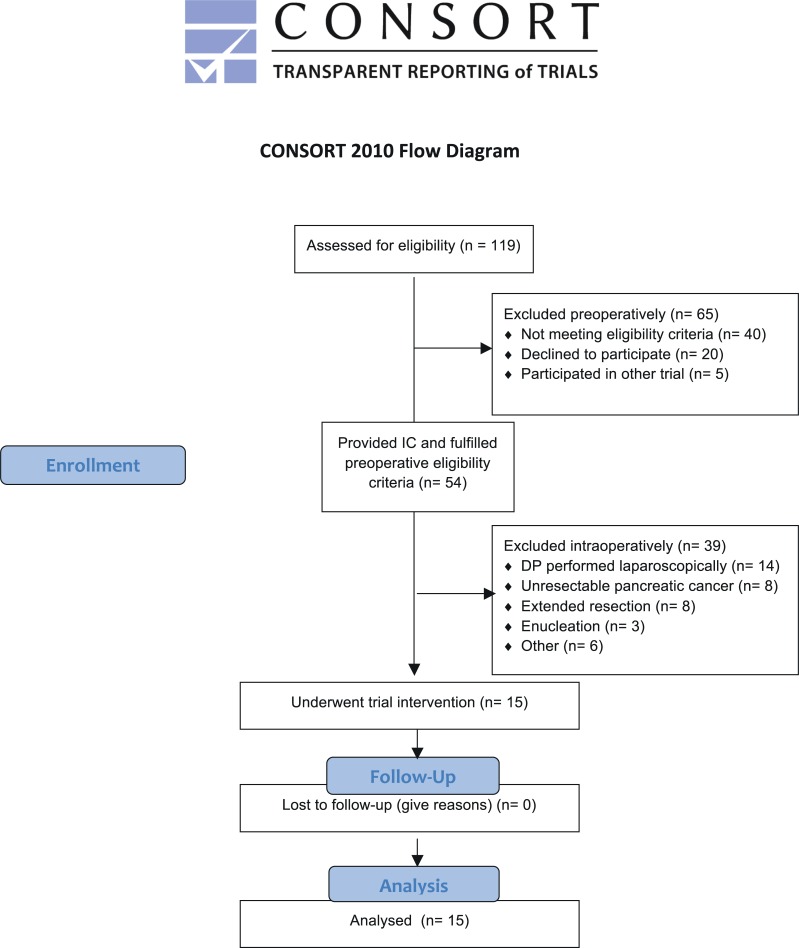
Study flow chart displaying the selection of the study cohort.

Between April 14^th^ 2015 and June 10^th^ 2016 119 patients scheduled for explorative laparotomy and distal pancreatectomy at the Department of General, Visceral, and Transplantation Surgery of the University Hospital Heidelberg were screened for inclusion in the study. A flowchart is provided in Fig 1. Patients undergoing laparoscopic surgery had to be excluded because the applicator of ETHICON OMNEX was not suitable for laparoscopic surgery. During the study period, the manufacturer of the investigational device stopped production and market distribution of the surgical sealant in general. Therefore, the planned number of 35 patients could not be accrued.

Finally, 15 patients (8 male, 7 female) with a median age of 63 years (41–80) were included and treated with the 2-OCA application. The indications were pancreatic ductal adenocarcinoma in nine cases, intraductal papillary mucinous neoplasms in two cases, two neuroendocrine tumors, one mucinous cystic neoplasm and one serous cystadenoma. Three patients underwent spleen-preserving distal pancreatectomy, whereas the rest underwent simultaneous splenectomy. 5 patients underwent extended resections, two patients underwent simultaneous atypical liver resections, one adrenalectomy, one simultaneous enucleation of a cystic lesion in the pancreatic head and one patient underwent multivisceral resection including subtotal gastrectomy, adrenalectomy and left hemicolectomy with colostomy. 2-OCA was applied as a single-layer in 8 patients and as double-layer in the remaining 7 patients. Further baseline characteristics are shown in Table 2.

**Table 2:** Baseline characteristics of the study population and intraoperative endpoints.

		n = 15	Additional information
Gender	male	8 (53.3%)	
	female	7 (46.7%)	
Age (years)		63 (41–80)	
BMI (kg/m^2)^		26.09 (16.41–33.56)	
Previous abdominal surgery		7 (46.7%)	appendectomy (n = 4), hysterectomy (n = 2), sigmoid resection (n = 1)
ASA	I	0 (0%)	
	II	12 (80%)	
	III	3 (20%)	
Cardiac comorbidities		3 (20%)	Coronary heart disease (n = 1), n. s. (n = 2)
Pulmonary comorbidities		4 (26.7%)	COPD (n = 3); asthma (n = 1)
Renal comorbidities		0 (0%)	
Hepatic comorbidities		2 (13.3%)	Liver metastases (n = 1); thrombosis of the portal venous system (n = 1)
Previous chemotherapy		1 (6,7%)	
Pathologic result			
	PDAC	9 (60%)	
	IPMN	2 (13.3%)	
	NET	2 (13.3%)	
	MCN	1 (6.7%)	
	SCN	1 (6.7%)	
Previous diabetes mellitus		1 (6.7%)	
Previous exocrine insufficiency		1 (6.7%)	
Distal pancreatectomy			
	spleen-preserving	3 (20%)	
	with splenectomy	12 (80%)	
Extended resections		5 (33.3%)	Atypical liver resection (n = 2), adrenalectomy (n = 1), simultaneous enucleation in the pancreatic head (n = 1), multivisceral resection including subtotal gastrectomy, adrenalctomy and left hemicolectomy with colostomy (n = 1)
Duration of surgery (min)		165 (115–258)	Lower quartile: 145; upper quartile: 204; 95% CI: 154.83–195.44
Estimated blood loss (ml)		600 (300–3000)	Lower quartile: 350; upper quartile: 750; 95% CI: 370.73–1022.60
Mode of application			
	single-layer	8 (53.3%)	
	double-layer	7 (46.7%)	
Pancreatic texture			
	Soft	8 (53.3%)	
	Moderate	3 (20.0%)	
	n. s.	4 (26.7%)	

Given are numbers (percent) for binary data or median (range) for continuous variables; BMI = body mass index; ASA = American Society for Anaesthesiologists; COPD = chronic obstructive pulmonary disease; PDAC = pancreatic ductal adenocarcinoma; IPMN = intraductal papillary mucinous neoplasm; NET = neuroendocrine tumour; MCN = mucinous cystic neoplasm; SCN = serous cystic neoplasm

Mean duration of surgery was 165 minutes (115–258) and the mean estimated blood loss was 600 ml (300–3000). In all patients two passive, intra-abdominal drains were placed close to the pancreatic remnant an in the left subphrenic region respectively.

In summary, a total of ten SAEs occurred in eight patients (one patient suffered three SAEs). There were no device-related adverse events and no mortality within 3 months after surgery. Five SAEs were direct surgical complications more specifically three cases of POPF and two cases of PPH. Three general complications fulfilled SAE criteria, of which two cases showed tumor infiltration of the pancreatic resection margin in the final pathological workup after tumor-free intraoperative frozen sections and one case of a vasovagal syncopation. Furthermore one SAE was classified as affecting the gastrointestinal tract (malnutrition after multivisceral resection) and one SAE affected the (sub-)cutaneous tissue (abdominal wall abscess after long-lasting percutaneous drainage). The SAEs and their consequences are detailed in Table 3.

**Table 3:** List of serious adverse events and their consequences.

Patient #	Diagnosis of SAE	Countermeasures	Outcome of SAE
1	PPH in the region of urinary bladder	Reoperation on POD 1 with evacuation of haematoma and control of bleeding	Recovered completely
4	PPH retroperitoneal in a patient after multivisceral resection	Angiography, reoperation on POD 9 with evacuation of haematoma and control of bleeding	Resolved with sequelae
4	Limited oral food intake and consecutive malnutrition in a patient after multivisceral resection	Supportive parenteral nutrition	Ongoing at the end of study (3 month follow-up)
18	Vasovagal syncopation after physical strain in an elderly patient	Hospitalization for diagnostics and observation without need for medical intervention	Recovered completely
4	Abscess of the abdominal wall after prolonged interventional drainage of POPF	Wound debridement and vaccum therapy	Recovered completely
35	POPF with intraabdominal fluid collection	Percutaneous CT-guided drainage	Recovered completely
69	Incomplete oncologic resection in final histologic specimen (R1-situation) after tumor-free intraoperative frozen section	Reoperation with completion pancreatectomy	Resolved completely
86	POPF	endoscopic intervention (injection of botox into the sphincter of oddi), endosonographic puncture of the intraabdominal fluid collection and finally percutaneous CT-guided drainage of the fluid collection; antibiotic therapy	Recovered completely
91	POPF with intraabdominal fluid collection	Reoperation on POD 14 with lavage, drainage of the fluid collection and antibiotic therapy	Recovered completely
99	Incomplete oncologic resection in final histologic specimen (R1-situation) after tumor-free intraoperative frozen section	Reoperation with completion pancreatectomy	Resolved completely

In total, there were six (40%) biochemical leaks and five (33.3%) clinically relevant cases of POPF (three grade B and two grade C). There were two cases (13.3%) of DGE (one grade A and one grade B) and two cases (13.3%) of PPH (one grade A and one grade B). Further complications included three cases (20%) of intra-abdominal abscess and three cases (20%) of surgical site infections. Relaparotomy was necessary in a total of five patients (33.3%), two due to PPH, two for completion pancreatectomy due to tumor infiltration of the pancreatic resection margin in the final pathological workup and one case for drainage of an intra-abdominal abscess. Percutaneous drainage was performed in four patients (26.7%). The median comprehensive complication index was 24.2 (range: 0–61; lower quartile: 13.62; upper quartile: 33.63; 95% CI: 17.42–32.71). There were neither cases of postoperative pancreatitis nor any cardiovascular complications (myocardial infarction, cerebrovascular accident, deep vein thrombosis or pulmonary embolism).

The median length of hospital stay was ten days (range: 5–76; lower quartile: 8; upper quartile: 20; 95% CI: 7.8–27.43) and four patients (26.7%) were readmitted to the hospital during the three-month follow-up period. No cases of postoperative new onset of exocrine or endocrine insufficiency occurred except of the two cases that underwent remnant pancreatectomy due to positive resection margins in final pathologic workup.

## Discussion

The main objective of this proof-of-concept study was to assess safety and feasibility of 2-OCA application to the pancreatic remnant after distal pancreatectomy. The application was easily performed without intraoperative problems during the application process and the sealant remained in the intended place as seen in those patients that necessitated relaparotomy. In addition, no device-related adverse events occurred in this study. Thus, the study intervention proofed to be feasible and seemed safe in the present cohort based on a small number of patients.

POPF remains a major concern after pancreatic surgery especially after distal pancreatectomy and pancreatic surgeons still lack a sufficient tool or technical modification to prevent leakage of the pancreatic stump. [23] Therefore, another aim of the study was to gather preliminary efficacy data for the study intervention regarding POPF rate to allow sample size calculation for a subsequent confirmatory trial. However, the planned number of 35 patients could not be accrued within this study because production and market distribution of the investigational device was stopped in general during the study period. Due to the low sample size of the current study, assessment of efficacy of the intervention is not reasonable because of the uncertainty of the results. However, with a clinically relevant POPF rate of 33.3% the current results are comparable to POPF rates after distal pancreatectomy reported in other trials or larger cohorts. More particularly, the rate of clinically relevant POPF was 20.5% in the DISPACT-trial, [18] 27.6% in the DISCOVER-trial [4] and in the FIABLE-trial assessing sealing with a fibrin collagen patch the rate was 27.4%. [5] More recently, a retrospective cohort study evaluating 2-OCA sealing together with a falciform ligament patch revealed a clinically relevant POPF rate of 36% in patients after distal pancreatectomy, which is even slightly higher as in the current study. [24].

Over the last decades, surgical researchers have tested multiple technical variations and different sealants in an attempt to reduce the occurrence of POPF after distal pancreatectomy. Hand-sewn closure was compared against stapled resection without differences in POPF rates. [13] Furthermore, various sealants, especially fibrin sealants showed promising results in smaller studies [25] but could not prove efficacy in larger randomized controlled trials. [5, 11] Corroborated by the pooled findings of these trials in meta-analyses, fibrin sealants cannot be recommended for routine use. [26, 27] Equally, constructing a pancreato-intestinal anastomosis on the pancreatic stump did not result in lower POPF rates but longer duration of surgery and is thus rarely performed. [28, 29] Another option of reinforcement of the resection surface is coverage by autologous tissue, which is mostly done using the falciform or teres ligament of the liver. A recent randomized controlled trial from the authors’ institution came to the result that POPF rates were not reduced but the need for reinterventions, reoperations and readmissions was less in the group with a teres ligament patch. [4] Furthermore, some trials have focused on optimization of pancreatic drainage over the papilla of Vater by prophylactic endoscopic stenting of the pancreatic duct [30] or in a more recent trial by preoperative endoscopic injection of botulinum toxin into the sphincter of Oddi. [31] However, stenting did not reduce the POPF rates in the randomized controlled trial and botulinum toxin injection showed promising results in the pilot study but a controlled trial is still missing for this approach. Thus, pancreatic surgeons are still lacking a reliable tool to effectively prevent POPF after distal pancreatectomy.

The current study has to be interpreted in the light of some limitations. First, the planned sample size of 35 patients was not reached, because the distribution of the investigational product was stopped during the recruitment period of the study. Thus, some rare device-related events may have been missed due to the low sample size and the planned evaluation of efficacy was not possible based on the current study. Second, an increasing number of distal pancreatectomies are nowadays performed by a minimally invasive approach (either laparoscopic or robotic) and to our knowledge there is no 2-OCA application device suitable for minimally invasive surgery, which limits the applicability of the intervention in modern surgical concepts. Nevertheless, the prospective and structured study design guarantees for high methodological and data quality of the current results.

## Conclusions

In conclusion, 2-OCA application to the pancreatic remnant seems feasible and safe based on limited data. The inadvertent early termination of the current study presents a major limitation, which made a judgement on preliminary efficacy of the intervention impossible. However, together with evidence from further recent studies, 2-OCA application does not seem to be the most promising approach to reduce POPF rates after distal pancreatectomy. Therefore, novel concepts for stump sealing or other preventive measures to reduce POPF rates after distal pancreatectomy should be the focus of future research projects.

## Supporting information

S1 Checklist TREND checklist. (PDF)

S1 Protocol Study protocol BOND. (PDF)

S1 Dataset BOND_full dataset. (XLSX)

## Abbreviations

2-OCA: 2-octyl cyanoacrylate
DGE: Delayed gastric emptying
ISGPF: International study group for pancreatic fistula
ISGPS: International study group of pancreatic surgery
POPF: Postoperative pancreatic fistula
PPH: Postpancreatectomy haemorrhage
SAE: Serious adverse event
