# Supplementary material for: 2-octyl cyanoacrylate sealing of the pancreatic remnant after distal pancreatectomy – A prospective pilot study
Source: PLoS One. 2018 Oct 16;13(10):e0205748. doi: 10.1371/journal.pone.0205748 (PMC6191135; doi:10.1371/journal.pone.0205748)
Supplement: S1 Protocol — (PDF) [file pone.0205748.s002.pdf]

CLINICAL TRIAL PROTOCOL

Trial title

Prospective open-label monocenter clinical trial to evaluate the safety and efficacy of a 2-octyl cyanoacrylate (2-OCA) bonding of the pancreatic remnant in the prevention of postoperative pancreatic fistula after distal pancreatic resection – the BOND-Trial

trial registration number

not yet registered

The information in this trial protocol is strictly confidential. It is for the use of the sponsor, investigator, trial personnel, ethics committee, the authorities, and trial subjects only. This trial protocol may not be passed on to third parties without the agreement of Markus K Diener, Head of the Study Center of the German Surgical Society, Im Neuenheimer Feld 110, 69120, Germany.

The BOND-trial will be conducted at the Department of General, Visceral, and Transplantation Surgery, University of Heidelberg, Im Neuenheimer Feld 110, 69120 Heidelberg

Funding/Logo/funding number

Funding by the *Heidelberger Stiftung Chirurgie*

ROLES AND RESPONSIBILITIES

Principal Investigator

Markus K. Diener, MD

Head of the Study Center of the German Surgical Society (SDGC)

Department of General, Visceral, and Transplantation Surgery

University of Heidelberg

Im Neuenheimer Feld 110, 69120 Heidelberg, Germany

Tel.: +49 6221 56 6986

Fax: +49 6221 56 6988

E-mail: markus.diener@med.uni-heidelberg.de

Coordinating Investigator

Felix J. Hüttner, MD

Study Center of the German Surgical Society (SDGC)

Department of General, Visceral, and Transplantation Surgery

University of Heidelberg

Im Neuenheimer Feld 110, 69120 Heidelberg, Germany

Tel.: +49 6221 56 6986

Fax: +49 6221 56 6988

E-mail: felix.huettner@med.uni-heidelberg.de

Biometrician

Dr. sc. hum. Thomas Bruckner

Institute of Medical Biometry and Informatics

University of Heidelberg

Im Neuenheimer Feld 305

69120 Heidelberg; Tel.: +49 6221 56 4371

bruckner@imbi.uni-heidelberg.de

Project Management

Inga Rossion, MD

Study Center of the German Surgical Society (SDGC)

Department of General, Visceral, and Transplantation Surgery

University of Heidelberg

Im Neuenheimer Feld 110

69120 Heidelberg

Telephone: +49 (0)6221 56 6984

e-mail: inga.rossion@med.uni-heidelberg.de

Data Management

R&P - Research & Public Relations

Prof. Dr. Dr. med. Reinhard Rychlik

Am Ziegelfeld 28

51399 Burscheid

Telephone: +49 (0) 2174 7152-24

Fax: +49 (0) 2174 7152-98

e-mail: info@randp.de

Quality Assurance

Clinical Monitoring

Study Center of the German Surgical Society (SDGC)

Department of General, Visceral, and Transplantation Surgery

University of Heidelberg

Im Neuenheimer Feld 110

69120 Heidelberg

Telephone: +49-(0)6221-56-36833

Fax: +49-(0)6221-56-33850

e-mail: marie-luise.memmer@med.uni-heidelberg.de

SAE-Management

Felix J. Hüttner, MD

Study Center of the German Surgical Society (SDGC)

Department of General, Visceral, and Transplantation Surgery

University of Heidelberg

Im Neuenheimer Feld 110, 69120 Heidelberg, Germany

Tel.: +49 6221 56 6986

Fax: +49 6221 56 6988

E-mail: felix.huettner@med.uni-heidelberg.de

<u>Sponsor</u>

Ruprecht-Karls-University Heidelberg, Medical Faculty

Represented by the University Hospital Heidelberg

and its Commercial Director:

Ms. Irmtraut Gürkan

Im Neuenheimer Feld 672

69120 Heidelberg

Tel.: +49 6221 56 7002

Fax: +49 6221 56 4888

E-mail: irmtraut.guerkan@med.uni-heidelberg.de

<u>Trial Committees</u>

<u>Data Safety Monitoring Board</u>

Prof. Dr. med. Volker Fendrich, Surgical Department, University Hospital Gießen/Marburg GmbH

e-mail: fendrich@med.uni-marburg.de

PD Dr. med. René Hennig, Surgical Department, Katharinenhospital Stuttgart

e-mail: r.hennig@klinikum-stuttgart.de

Prof. Dr. med. Christoph M. Seiler, MSc (Clinical Epidemiology), Surgical Department, Josephs-Hospital Warendorf

e-mail: c.seiler@jhwaf.de

<u>Steering committee</u>

Prof. Dr. med. Dr. h.c. mult. Markus W. Büchler, Medical Director of the Department of General, Visceral and Transplantation Surgery, University of Heidelberg, Heidelberg, Germany

PD Dr. med. Markus K. Diener, Department of General, Visceral, and Transplantation Surgery, University of Heidelberg, Heidelberg, Germany

Dr. sc. hum. Thomas Bruckner, Institute of Medical Biometry and Informatics (IMBI), University of Heidelberg, Heidelberg, Germany

SIGNATURE PAGE

The present trial protocol was subject to critical review and has been approved in the present version by the persons undersigned. The information contained is consistent with:

- The current risk-benefit assessment of the medical device
- the moral, ethical and scientific principles governing clinical research as set out in the latest relevant version of the Declaration of Helsinki and the applicable legal and regulatory requirements.

The investigators will be supplied with details of any significant or new findings including adverse events.

PD Dr. Markus K. Diener		
Principal Investigator<br>Representative of the Sponsor	Signature	Date

TABLE OF CONTENTS

SYNOPSIS 8
FLOW CHART 10
ABBREVIATIONS 11
1. INTRODUCTION 12
1.1 SCIENTIFIC BACKGROUND 12
1.2 TRIAL RATIONALE 13
1.3 OBJECTIVES 13
1.4 TRIAL DESIGN 13
1.5 TRIAL DURATION AND SCHEDULE 13
2. TRIAL CONDUCT 14
2.1 ELIGIBILITY CRITERIA 14
2.1.1 Number of patients and trial centers 14
2.1.2 Criteria for withdrawal of patients 15
2.2 INTERVENTION(S) 15
2.2.1 Investigational Device 15
2.2.2 Description of trial interventions 16
2.2.3 Benefits and risks of trial interventions 16
2.2.4 Procedures for minimization of risks 18
2.2.5 Assignment of intervention and blinding 18
2.3. OUTCOMES/ENDPOINTS 19
2.4. PATIENT SCHEDULE AND DOCUMENTATION 19
2.4.1 Description of trial visits 19
2.5 PLAN FOR FURTHER TREATMENT OF THE PATIENTS AFTER TERMINATION OF THE TRIAL 22
3. DATA MANAGEMENT 22
4. STATISTICAL PROCEDURES 23
4.1 SAMPLE SIZE CALCULATION 23
4.2 ANALYSIS VARIABLES AND STATISTICAL METHODS 23
5. QUALITY ASSURANCE 23
5.1 CLINICAL DATA MONITORING 23
5.2 ASSESSMENT OF SAFETY 24
5.2.1 Data safety and monitoring board (DSMB) 28
5.3 STEERING COMMITTEE 28
5.4 RECORD RETENTION AND DIRECT ACCESS TO SOURCE DATA/DOCUMENTS 28
6. DEVICE ACCOUNTABILITY 29
7. ETHICAL AND LEGAL ASPECTS 29
7.1 PREMATURE TERMINATION OF THE TRIAL 30
7.2 PROTOCOL APPROVAL AND AMENDMENTS 30
7.3 RESPONSIBILITIES OF INVESTIGATOR 31
8. AGREEMENTS 31
8.1 FINAL REPORT 31
8.2 FINANCING OF THE TRIAL 31

8.3 DISSEMINATION 31

8.4 TRANSLATIONAL RESEARCH 32

9. REFERENCES 32
10. DECLARATION OF INVESTIGATOR 35
APPENDICES 36
***APPENDIX I: OMNEX INSTRUCTIONS FOR USE (EFFECTIVE JULY 2014)*........ 36**

APPENDIX II: FDA'S SUMMARY OF SAFETY AND EFFECTIVENESS DATA 40

***APPENDIX III: SAE REPORTING FORM OF THE BFARM*........ 63**

SYNOPSIS

**Investigators**	Markus K. Diener & Felix Hüttner, MD<br>Study Center of the German Surgical Society (SDGC)<br>Dept. of General, Visceral, and Transplantation Surgery<br>University of Heidelberg,<br>Im Neuenheimer Feld 110, 69120 Heidelberg, Germany<br>Email: markus.diener@med.uni-heidelberg.de<br>Phone: +49 6221 56 6986, Fax: +49 6221 56 6988
**Title of study**	Prospective open-label monocenter clinical trial to evaluate the safety and efficacy of a 2-octyl cyanoacrylate (2-OCA) bonding of the pancreatic remnant in the prevention of postoperative pancreatic fistula after distal pancreatic resection – the BOND-Trial
**Condition**	Patients undergoing distal pancreatic resection for various underlying diseases
**Objective(s)**	To evaluate the safety and preliminary efficacy of an intraoperative 2-OCA application for the prevention of pancreatic fistula
**Intervention(s)**	Experimental intervention: Intraoperative 2-OCA application on pancreatic remnant in distal pancreatic resection (DP)<br>Follow-up per patient: 3 months<br>Duration of intervention per patient: 5 min application of the surgical sealant<br>Duration of complete operation: ~ 120-240 min
**Key inclusion and exclusion criteria**	Key inclusion criteria:<br>• Patients scheduled for elective distal pancreatic resection (DP)<br>• ≥ 18 years of age<br>• Written informed consent<br>Key exclusion criteria:<br>• Haemoglobin< 10 g/dl<br>• Bilirubin > 3 times ULN<br>• AST or ALT > 4 ULN<br>• INR > 1.7<br>• Creatinine clearance < 30 ml/min (estimated by Cockcroft-Gault)<br>• Serious cardiovascular disease (e.g. myocardial infarction in the last 12 months, congestive heart failure NYHA III/IV, unstable angina pectoris)<br>• Liver cirrhosis (of any Child-Pugh grade)<br>• ASA score > III<br>• Immunosuppressive therapy (cortison ≥ 40 mg/d or equivalent; azathioprin)<br>• Pregnancy or lactation<br>• Drug trial participation within 30 days before screening visit<br>• Understanding or language problems<br>• Inability to comply with study and/or follow-up procedures<br>• Allergy or known intolerability to 2-octyl cyanoacrylate, butyl-lactoyl-cyanoacrylate or formaldehyde<br>• Any condition which could result in an undue risk for the patient in the opinion of the investigator
**Outcome(s)**	Endpoints:<br>Primary safety endpoint:<br>• Frequency of serious adverse events and device-related adverse events<br>Primary efficacy endpoint:<br>• Occurrence of a postoperative pancreatic fistula (according to the ISGPF definition[1]) within 30 days after the index operation<br>Secondary endpoints:

	• Surgical:<br>o Delayed gastric emptying (according to the ISGPS definition) [2]<br>o Postpancreatectomy hemorrhage (according to the ISGPS definition) [3]<br>o Postoperative pancreatitis<br>o Intra-abdominal abscess or fluid collection<br>o Relaparotomy<br>o Burst abdomen<br>o Wound infection<br>• Cardiovascular:<br>o Perioperative myocardial infarction<br>o Perioperative cerebral vascular incident<br>o Perioperative deep vein thrombosis<br>• Pulmonary:<br>o Perioperative lung embolism<br>• 30-day mortality<br>• Operation time<br>• Intraoperative blood loss<br>• Postoperative hospital stay<br>Assessment of safety: Patients will be closely monitored for the occurrence of any (serious) adverse events. Adverse events will be categorized in surgical, cardiovascular, pulmonary, urinary and others. Furthermore it will be defined if adverse events are device-related or not.
**Study type**	A monocenter open label prospective proof of concept trial in a single arm study design at the development stage (according to the IDEAL recommendations [4])
**Statistical analysis**	Descriptive statistical analysis will be conducted.
**Sample size**	To be assessed for eligibility:**(n = 50)**<br>To be allocated to trial: **(n = 35)**<br>To be analyzed: **(n = 30)**
**Trial duration**	First patient in to last patient out (months): 12 months<br>Duration of the entire trial (months): 24 months<br>Recruitment period (months): 6 months<br>Trial report completed (months): 6 months after last-patient-out
**Participating centers**	Dept. of General, Visceral, and Transplantation Surgery, University of Heidelberg

FLOW CHART

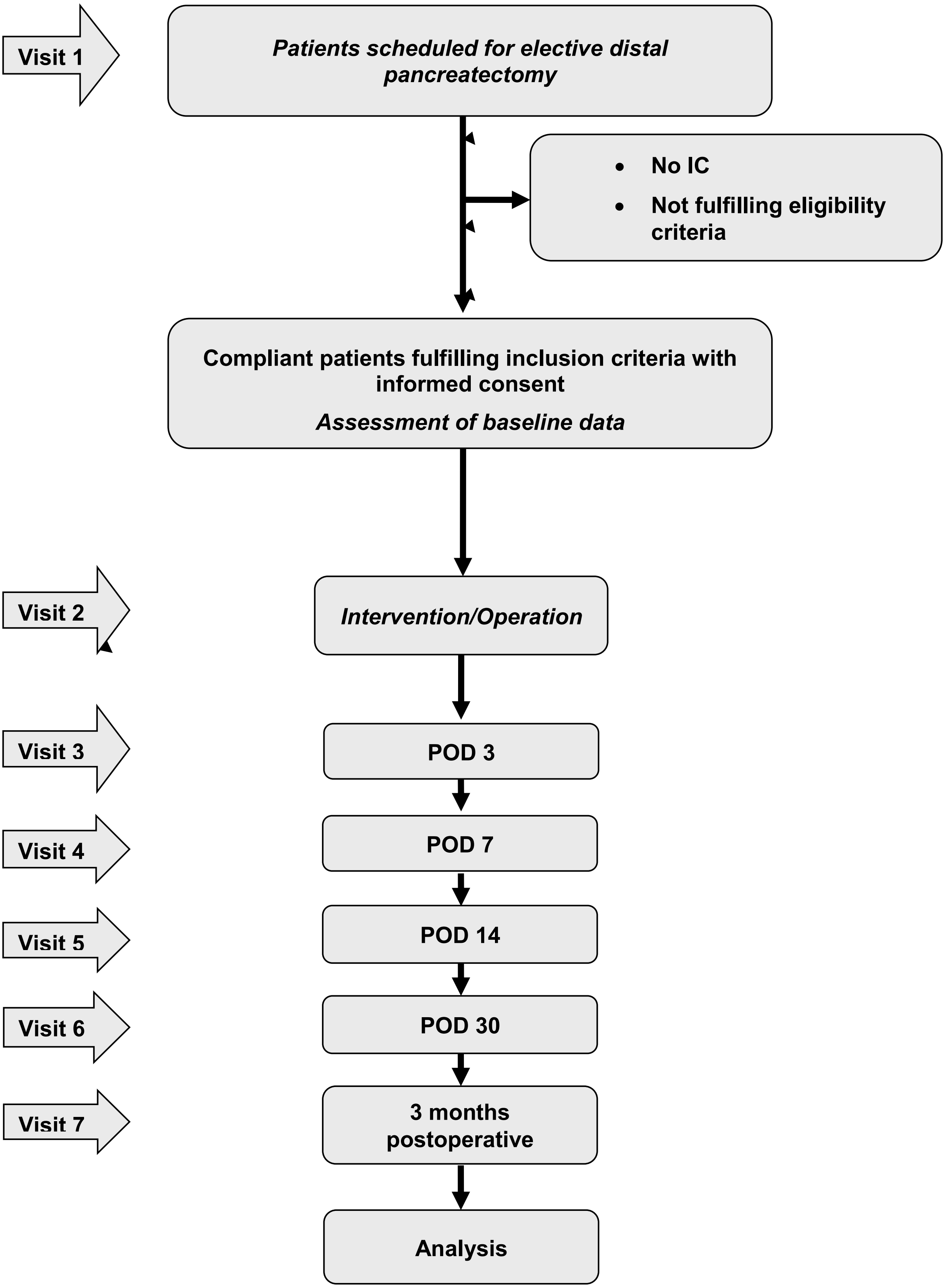

Visit 1
Patients scheduled for elective distal pancreatectomy
No IC
Not fulfilling eligibility criteria
Compliant patients fulfilling inclusion criteria with informed consent
Assessment of baseline data
Visit 2
Intervention/Operation
Visit 3
POD 3
Visit 4
POD 7
Visit 5
POD 14
Visit 6
POD 30
Visit 7
3 months postoperative
Analysis

ABBREVIATIONS

**2-OCA**	2-Octyl Cyanoacrylate
**AE**	Adverse Event
**ALT**	Alanine Aminotransferase
**AST**	Aspartate Aminotransferase
**BLCA**	Butyl Lactoyl Cyanoacrylate
**CI**	Confidence Interval
**CRF**	Case Report Form
**CRP**	C-Reactive Protein
**CV**	Curriculum vitae
**DP**	Distal pancreatectomy
**DSMB**	Data Safety Monitoring Board
**EC**	Ethics Committee
**FPI**	First Patient In
**FU**	Follow-up
**GCP**	Good Clinical Practice
**ICH**	International Conference on Harmonization of Technical Requirements for Registration of Pharmaceuticals for Human Use
**IEC**	Independent Ethics Committee
**IMBI**	Institute of Medical Biometry and Informatics
**ITT**	Intention-To-Treat
**ISF**	Investigator Site File
**KKS**	Coordination Centre for Clinical Trials
**LPI**	Last Patient In
**LPO**	Last Patient Out
**PD**	Pancreaticoduodenectomy
**POD**	Post Operation Day
**POPF**	Postoperative Pancreatic Fistula
**QLQ**	Quality of Life Questionnaire
**QoL**	Quality of Life
**RCT**	Randomized Controlled Trials
**RR**	Relative Risk
**SAE**	Serious Adverse Event
**SDGC**	Study Center of German Surgical Society
**SDV**	Source Data Verification
**SOP**	Standard Operating Procedure
**TMF**	Trial Master File
**USADE**	Unanticipated Serious Adverse Device Effect
**V**	Visit

1. INTRODUCTION

In Germany there are about 15,000 newly diagnosed neoplasms of the pancreas each year.[5] Exact numbers for benign or borderline lesions do not exist. In 2011 aproximately 11,800 partial resections of the pancreas were performed in German hospitals.[6]

Pancreatic surgery is complex from a diagnostic, surgical and perioperative point of view. Centralization of pancreatic surgery in specialized institutions has led to acceptable mortality rates below 5%.[7-9] Moreover, standardization of surgical and perioperative care in these centers of expertise is a prerequisite for low morbidity rates.[7]

Postoperative pancreatic fistula (POPF) still represents the most common postoperative morbidity in pancreatic surgery and can profoundly affect patient recovery and outcome. The incidence of POPF reported in the literature varies widely with overall rates between 0% and 24%.[10] After pancreaticoduodenectomy (PD) POPF rates range from 10% to 15% in different studies, and usually occur at a higher rate of about 10% to 30% after DP respectively.[11] In a large multicenter RCT conducted by the SDGC comparing stapler vs. hand-sewn closure of the pancreas after DP, the overall POPF rate was 30% in 352 analyzed patients, coinciding well with the rates in the literature.[12]

In-hospital mortality due to POPF or subsequent complications occur in up to 14%,[13] reaching up to 33% in high-risk subgroups.[14]

Pancreatic surgeons still lack an effective strategy to reduce the rates of POPF. Therefore, further research on the prevention of POPF is obligatory.

1.1 SCIENTIFIC BACKGROUND

In current routine practice, after DP the pancreatic remnant is usually closed by either the use of a stapler or by hand-suture with conventional surgical sutures without any further measures. Several surgical techniques and technical modifications have been proposed in an attempt to reduce fistula rates in pancreatic surgery.[15] For instance, different types of fibrin sealants have been evaluated in their potential to reduce the occurrence of POPF, but none of them has been proven effective so far.[16, 17] A recent systematic review and meta-analysis of Orci et al. [18] concluded that “fibrin sealants cannot be recommended for routine clinical use in the setting of pancreatic surgery”. Also, mesh reinforcements of the pancreaticojejunal anastomosis have provided no significant benefit in the reduction of POPF.[19]

Compared to fibrin sealants, the medical glue 2-OCA is also easily applicable to the resection surface and will not be degraded by aggressive pancreatic enzymes due to its long-lasting tissue bonds. Cyanoacrylate is an acrylic resin that rapidly polymerizes in the

presence of water, forming, long, strong bonds that join surfaces together. The compound 2-OCA is a nontoxic bacteriostatic medical glue that has been widely used to approximate skin edges.[20] Recently, ETHICON™ OMNEX™ a new surgical sealant, consisting of a blend of 2-OCA and butyl-cyanoacrylate (BLCA), has been approved by the FDA and has been CE-certified for the intracorporal use in vascular surgery.

In 2013,Barakat et al. [21] have published their first results on the topical application of 2-OCA to the pancreaticojejunal anastomosis after PD. They reported a highly significant reduction of POPF for the 2-OCA group compared to patients without 2-OCA application. The rate of POPF in the 2-OCA group was 3.5% compared to 36% in the group without 2-OCA application. Currently, no further evidence on the application of 2-OCA in pancreatic surgery is available in the literature.

1.2 TRIAL RATIONALE

Based on the results of Barakat et al. [21], topical 2-OCA application promises a substantial benefit in the prevention of POPF. Currently, there are only data available on the topical application to the pancreaticojejunal anastomosis after PD. Therefore, we intend to expand this promising technique to further pancreatic interventions. DP shows even higher rates of POPF compared to PD, thus, there may be a larger benefit for prevention of POPF in this indication.

Based on these facts, we conduct this trial in a proof-of-concept design to further develop the technique and to evaluate safety and preliminary efficacy in these indications.

1.3 OBJECTIVES

The main objective of this study is to evaluate the safety of a 2-OCA application to the pancreatic remnant after distal pancreatectomy. Therefore all serious adverse events will be closely monitored. If the intervention proofs to be safe under these conditions, a multicenter RCT will be planned to evaluate the efficacy of this new and promising technique.

As primary efficacy parameter, the rate of POPF will be assessed.

1.4 TRIAL DESIGN

BOND is amonocenter open label prospective proof of concept and safety trial in a single arm study design at the development stage (according to the IDEAL recommendations [4])

1.5 TRIAL DURATION AND SCHEDULE

The duration of the trial for each patient is planned to be 3 months. The duration of the overall trial is expected to be 2 years, including prearrangement and analysis.

2. TRIAL CONDUCT

2.1 ELIGIBILITY CRITERIA

Key inclusion criteria:

- Patients scheduled for elective distal pancreatic resection (DP)
- ≥ 18 years of age
- Written informed consent

Key exclusion criteria:

- Haemoglobin< 10 g/dl
- Bilirubin > 3 times ULN
- AST or ALT > 4 ULN
- INR > 1.7
- Creatinine clearance < 30 ml/min (estimated by Cockcroft-Gault)
- Serious cardiovascular disease (e.g. myocardial infarction in the last 12 months, congestive heart failure NYHA III/IV, unstable angina pectoris)
- Liver cirrhosis (of any Child-Pugh grade)
- ASA score > III
- Immunosuppressive therapy (cortison ≥ 40 mg/d or equivalent; azathioprin)
- Pregnancy or lactation
- Drug trial participation within 30 days before screening visit
- Understanding or language problems
- Inability to comply with study and/or follow-up procedures
- Allergy or known intolerability to 2-OCA, butyl-lactoyl cyanoacrylate or formaldehyde
- Any condition which could result in an undue risk for the patient in the opinion of the investigator

2.1.1 Number of patients and trial centers

BOND will be conducted as a monocenter proof-of-concept and safety trial, therefore no actual sample size calculation was performed. A total of 35 patients is planned to be included into the trial. From our previous RCT on DP and from a review of the literature, there is good evidence that the rate of POPF after conventional DP is 30%.[12] Thus, we would expect the occurrence of about 10 cases of POPF without application of the sealant which should be reduced by the application of the 2-OCA sealant. Therefore, a number of 35 was judged sufficient for a preliminary evaluation of safety and applicability by the investigators in this early phase of the new technique. BOND will be conducted as a monocenter trial at the Department of General, Visceral, and Transplantation Surgery at the University of Heidelberg, Heidelberg, Germany. If the trial intervention proofs to be safe, a multicenter RCT is planned with a sample size calculation based on the preliminary efficacy results (frequency of POPF).

2.1.2 Criteria for withdrawal of patients

Patients are free to leave the trial at any time and without giving reasons for their decision. Subjects may be withdrawn from the trial for the following reasons: (a) At their own request or (b) If, in the investigator's opinion, continuation of the trial would be detrimental to the subject's well-being. In case of (b), the reason for withdrawal must be recorded in the CRF and in the patient's medical records.

Patients that were included into the trial but the designated intervention could not be performed due to any reason (e.g. DP was not feasible upon abdominal exploration) will be replaced by consecutive patients until the planned sample size is reached.

2.2 INTERVENTION(S)

2.2.1 Investigational Device

The name of the investigational device is ETHICON™ OMNEX™ Surgical Sealant. The device is manufactured for ETHICON™ by Closure Medical Corp., Raleigh, North Carolina, 27616. ETHICON™ is a division of Johnson & Johnson Medical Limited. Distribution in Germany is done by Johnson & Johnson Wound Management, Oststraße 1, 22844 Norderstedt, Germany.

ETHICON™ OMNEX™ Surgical Sealant is a synthetic tissue sealant consisting of a blend of two monomers, 2-OCA and butyl-lactoyl-cyanoacrylate (see "Instructions for Use" in the Appendix). The liquid formulation is contained in a crushable glass ampoule, which is housed in a single-use delivery device. The formulation is passed through a porous disc containing an initiator, mixed in a chamber, and delivered through a cannula. When polymerization is complete, a film is formed that mechanically interlocks the tissue and/or non-biological materials (i.e. synthetic graft sutures, staples, clips) and creates a flexible physical seal, independent of the body's clotting mechanism. ETHICON™ OMNEX™ Surgical Sealant begins to polymerize immediately on mixing with the initiator and forms a physical seal within 2 minutes after application. ETHICON™ OMNEX™ Surgical Sealant has been formulated to provide a strong physical seal that remains in place beyond the time required for natural healing, and eventually degrades via hydrolytic chain scission (over approximately 36 months), breaking down into smaller absorbable fragments.

The sterile, non-pyrogenic device is provided as a packaged single-use applicator and stored at room temperature.

ETHICON™ OMNEX™ is a CE marked and certified device. It conforms to the essential requirements of the Medical Device Directive 93/42/EEG and was tested in conformity to the ISO 10993 series for biocompatibility of a long-term implantable medical device.

Further information about the investigational device including a summary of preclinical and clinical testing can be found in the investigator's brochure ("Handbuch des klinischen Prüfers"), provided by the SDGC.

2.2.2 Description of trial interventions

2-OCA in DP: After routine resection of the pancreatic tail and/or body the remnant will be closed according to local standards [22] by direct suture of the pancreatic duct with a non-reabsorbable surgical suture (e.g. Novafil 4-0) and closure of the pancreatic tissue with absorbable sutures (e.g. PDS 5-0) in fish-mouth technique. After the conventional closure, a thin layer of the 2-OCA surgical sealant will be applied to the sutured surface of the pancreatic remnant (not exceeding 4 ampoules). The surrounding area will be covered with sterile surgical gauzes to avoid contact of the sealant to other tissue not intended to get in contact with the sealant. Before application, the surface of the pancreatic remnant will be patted dry with a sterile gauze, to assure direct contact of the sealant to the tissue as described in the directions for use of the product. No additional covering of the pancreatic remnant will be conducted. Additional sutures of the cut surface for reasons of hemostasis will be allowed. After polymerization of at least 2-3 minutes the operation will be continued in a routine manner.[22] Perioperative treatment will not be changed by the trial intervention and will be conducted according to local standards.

2.2.3 Benefits and risks of trial interventions

ETHICON™ OMNEX™ has been tested extensively for biocompatibility, cytotoxicity, intracutaneous reactivity, dermal sensitization, acute toxicity, pyrogenicity, hemolysis, mutagenicity, etc. Furthermore, various animal studies including implantation studies up to 24 months have been performed. The tests were in accordance with ISO 10993 series and were appropriate for an implant device that is in permanent contact with tissue (>30 days). All the results indicated that the materials and processes used to manufacture ETHICON™ OMNEX™ Surgical Sealant and the delivery system are biocompatible and suitable for their intended use. The FDA's "Summary of Safety and Effectiveness Data" is provided in the appendix.

In addition to the preclinical testing, ETHICON™ OMNEX™ has been examined in a clinical feasibility study,[23] a RCT[24] and a prospective, single-arm, multicenter trial[25] of vascular surgery prior to its approval. The clinical trials showed no unanticipated adverse device effects. The complication profile was typical for patients undergoing vascular surgical procedures and did not raise any safety concerns. Furthermore, the number of patients with at least one vascular or bleeding complication was significantly reduced in the group treated with ETHICON™ OMNEX™, although the study was not powered for this analysis. The

(safety) results of the clinical trials are also given in the “Summary of Safety and Effectiveness Data” in the appendix.

Moreover, there are several reports on observed beneficial effects in “internal” use of 2-OCA sealants: e.g. heart surgery: ventricular wall ruptures;[26] thoracic surgery: closure of air leaks;[27] vascular surgery: embolization of aneurysms; endoscopy: bleeding varices of the esophageus and gastric fundus.[28-32] These reports show that internal use of 2-OCA is a safe procedure. We have conducted a literature search in Medline, identifying all available publications on the internal use of 2-OCA yielding 11 reports accounting for 473 patients. Overall, there have been only mild adverse events observed in 213 patients, mainly mild abdominal pain and transient fever resolving with conservative treatment in 2-4 weeks (see Table 1). In 3 % of the patients treated with 2-OCA, moderate AEs have been reported.

	**Author & year**	**n**	**Follow-Up**	**Indication/Intervention**	**mild AEs**	**moderate/serious AEs**
1	Wang J 2013	77	3 years	gastric varices: transhepatic vs. Endoscopic injection of 2-OCA	58 (fever + abdominal pain)	moderate AEs: 3 (pulmonary embolism, portal vein thrombosis) deaths: 0
2	Barakat O 2012	124 (75 2-OCA)	not stated	Prevention of POPF after PD by topical application of 2-OCA to the anastomosis	0	moderate AEs: 3 (cerebral vascular incidents, small bowel obstruction) deaths: 0
3	Binmoeller KF 2011	30	193d (Mean)	transesophageal EUS guided treatment of fundal varices with 2-OCA	no procedure related complications	no mortality
4	Tian X 2011	71	24.2 months	transhepatic variceal embolization with 2-OCA	62 (fever, abdominal pain + bacteremia)	moderate Aes: 5 (portal vein thrombosis, ulcer) deaths: 0
5	Carr JA 2011	7	up to 12 months	intracorporal application for air leak after lung resectioin	1 re-air-leak	no signs of toxicity deaths: 0
6	Lukish J 2010	7	12-20 months	closure of gastrocutaneous fistulas in children	no complications	no mortality
7	Carnero Alcázar M 2009	20	17.3 months (survivors)	sutureless surgery (2-OCA) for post-infarction free wall rupture	no 2-OCA related complications	deaths 4 due to cardiogenic shock and re ventricular rupture (not related to 2-OCA)
8	Zhang CQ 2009	92	37 m median	transhepatic varices embolization with 2-OCA	51 (fever + abdominal pain)	pulmonary embolism 1 deaths: 1 due to puncture-site bleeding
9	Zhang CQ 2008	52/50	24 m median	transhepatic variceal embolization with 2-OCA vs. Band ligation	35 (fever + abdominal pain)	moderate 3 (sepsis + portal vein thrombosis)
10	Aziz O 2007 (only Abstract)	17	not stated	2-OCA to control bleedings and air-leaks close to coronary anastomoses in cardiac surgery	no reported toxicity	no mortality
11	Rengstorff DS 2004	25	11+-4 m mean	endoscopic injection of 2-OCA into gastric varices	7 (fever + abdominal pain)	no mortality
		473			213 (45%)	15 (3%) (+ 5 deaths not related to 2-OCA)

Table 1: Adverse events of intracorporal application of 2-OCA (including intravascular use)

If intravascular application (for gastric and esophageal varices) is excluded, only 3 complications possibly related to intra-corporal 2-OCA application were identified in 119 patients (see Table 2).

	Author & year	n	Follow-Up	Indication/Intervention	mild AEs	moderate/serious AEs
2	Barakat O 2012	124 (75 2-OCA)	not stated	Prevention of POPF after PD by topical application of 2-OCA to the anastomosis	0	moderate AEs: 3 (cerebral vascular incidents, small bowel obstruction) deaths: 0
5	Carr JA 2011	7	up to 12 months	intracorporal application for air leak after lung resection	1 re-air-leak	no signs of toxicity deaths: 0
7	Carnero Alcázar M 2009	20	17.3 months (survivors)	sutureless surgery (2-OCA) for post-infarction free wall rupture	no 2-OCA related complications	deaths 4 due to cardiogenic shock and re ventricular rupture (not related to 2-OCA)
10	Aziz O 2007 (only Abstract)	17	not stated	2-OCA to control bleedings and air-leaks close to coronary anastomoses in cardiac surgery	no reported toxicity	no mortality
		119			no reported complications or toxicity; 1 re-air-leak	3 (2,5%) + 4 deaths not related to 2-OCA

Table 2: Adverse events of intracorporal non-vascular application of 2-OCA

The intended benefit is an effective sealing of the pancreatic remnant after routine closure by sutures and/or staples. The aim of this sealing is to reduce the rate of POPF, which represents one of the most frequent and potentially most hazardous complications after DP.

A conceivable adverse event could be unintended adhesions of other tissues (e.g. bowels) due to unintended contact of the surgical sealant to these tissues. This will be avoided through covering of the surrounding area with sterile gauzes during the application and polymerization process. The sterile gauzes will be removed after the sealant has fully polymerized.

2.2.4 Procedures for minimization of risks

For minimization of potential risks, study specific handling instructions for the investigational device have been defined in the investigator’s brochure. All investigators are obliged to read the investigator’s brochure (“Handbuch des klinischen Prüfers”) as well as the study protocol prior to trial start. Furthermore, investigators will be trained at a pre-trial study visit.

The study will be monitored to ensure the identification, documentation and analysis of all adverse events and compliance with the protocol. Furthermore, it will be ascertained, that the terms of the IEC to protect safety and rights of all subjects, and all other applicable regulations will be followed.

A Data Safety and Monitoring Board will be implemented for ongoing safety monitoring of the study.

2.2.5 Assignment of intervention and blinding

The primary aim of the BOND-trial is the evaluation of safety of the intervention. All participants will receive treatment with the 2-OCA surgical sealant and will be closely monitored for potential serious adverse events. Therefore patients and outcome assessors will not be blinded in this setting.

2.3. OUTCOMES/ENDPOINTS

Safety objectives: The incidence of all adverse events (AE) and serious adverse events (SAE) will be closely monitored and evaluated. All (S)AE and intervention related side effects will be documented on the specific forms (CRF, SAE-form).

Endpoints:
Primary safety endpoint:

- Frequency of serious adverse events and device-related adverse events (as categorized below)

Primary efficacy endpoint:

- Occurrence of a postoperative pancreatic fistula (according to the ISGPF definition [1]) within 30 days after the index operation.

Secondary endpoints:

- Surgical:
  - Delayed gastric emptying (according to the ISGPS definition) [2]
  - Postpancreatectomy hemorrhage (according to the ISGPS definition) [3]
  - Postoperative pancreatitis
  - Intra-abdominal abscess or fluid collection
  - Relaparotomy
  - Burst abdomen
  - Wound infection
- Cardiovascular:
  - Perioperative myocardial infarction
  - Perioperative cerebral vascular incident
  - Perioperative deep vein thrombosis
- Pulmonary:
  - Perioperative lung embolism
- 30-day mortality
- Operation time
- Intraoperative blood loss
- Postoperative hospital stay

2.4. PATIENT SCHEDULE AND DOCUMENTATION

2.4.1 Description of trial visits

Before acquisition of any data, patients will be informed about the trial and all trial-specific procedures (including data handling and data protection) by one of the investigators.

Determination of outcome measures

Visit 1

Baseline data: If a patient has given informed consent and all eligibility criteria are fulfilled, then the following baseline data will be documented.
Demographic data:

- Gender [m/f]
- Age [years]
- Height [cm]

- Weight [kg]

Baseline clinical data:

- Previous abdominal surgery
- Relevant comorbidities (cardiac, pulmonary, renal, hepatic)
- Smoking (former or current, cigarettes per day, years of consumption)
- Alcohol abuse (former or persistent, years of abuse)
- American Society of Anesthesiologists (ASA) Category (I to III according to the anesthesiologist's protocol)
- Previous radio-/chemotherapy in the last 6 months
- Laboratory tests including complete blood count, INR, bilirubin, AST, ALT, Creatinine clearance < 30 ml/min (estimated by Cockcroft-Gault), albumin, CRP
- Pregnancy test

Pancreas-specific medical history:

- Indication for planned surgery (pancreatic carcinoma, chronic pancreatitis, cystic neoplasia, neuroendocrine tumor, other)
- Pre-existing diabetes mellitus
- Pre-existing exocrine insufficiency

Sampling of 20 ml of blood for translational research in the context of a routine blood sample

Visit 2:

The following operative data are documented:

- Performed surgical intervention in detail:
  - DP
  - Additional resections like lymphadenectomy, splenectomy or extrapancreatic resections normally not included in the above mentioned techniques
  - Operation time defined as the interval from first incision of the skin until last knot of the skin suture, last skin staple respectively
  - Intraoperative blood loss as estimate from the anaesthesia protocol
  - Exact description of the application of 2-OCA (one layer, two layers, application problems, etc.)
  - Description of any problems during the application process (e.g. adhesive did not stick to the intended area, contact of the adhesive with other tissue, etc.)
  - Consistency of the pancreas: soft, medium, hard

  If any sealants or other procedures not mentioned in the study protocol are used, this has to be documented in the CRF.
- (Serious) adverse events

Visit 3, 4 and 5

On day 3, 7 and 14 after operation, the patients will be visited by study personnel. Furthermore, medical records of the patients will be checked to assess the primary and secondary endpoints. If the patients are dismissed before day 14 after the initial operation, Visit 5 will be performed within 36 hours prior to discharge.

- Postoperative occurrence of pancreatic fistula
  - Assessment by amylase activity in the drain fluid on postoperative day 3 according to the ISGPF definition [1]
- Complete drainage output assessment, including date/time of drainage removal, daily volume of drainage output at the specified visits, assessment of amylase and lipase activity in drainage fluid on day 3 and day 7/14, if drainage is still in place or new interventional drainage has been placed, date/time of placement of interventional drains.
- Assessment of perioperative secondary endpoints described above (see 2.3.2)
- (Serious) adverse events

Visit 6

On day 30 after the initial operation patients will be contacted by telephone and asked for the occurrence of further complications in a standardized interview. In case, further information is required, the treating physician will be contacted, if patients gave their consent to this. The following data are documented:

- Postoperative occurrence of pancreatic fistula (anamnestic)
- Assessment of the following perioperative secondary endpoints:
  - Delayed gastric emptying (according to ISGPS definition) [2]
  - Postpancreatectomy hemorrhage (according to the ISGPS definition) [3]
  - Postoperative pancreatitis
  - Intraabdominal abscess
  - 30-day mortality
  - Relaparotomy
  - Burst abdomen
  - Wound infection
  - Perioperative sepsis
  - Perioperative lung embolism
  - Perioperative myocardial infarction
  - Perioperative stroke
  - Perioperative deep vein thrombosis
- (Serious) adverse events

Visit 7

3 months after the index operation, the patients will be contacted again by telephone to evaluate the occurrence of further complications. A standardized telephone interview like detailed above (Visit 6) will be conducted.

Table 2 gives an overview of trial visits:

**Visit**	**1**	**2**	**3**	**4**	**5**	**6**	**7**
	Scree-ning	*Day of OP*	*Day 3 post OP*	*Day 7 post OP*	*Day 14 post OP/ Hos-pital dis-charge*	*Day 30 post OP*	*3 month post OP*
Demographics, baseline clinical data and laboratory tests	X						
Eligibility criteria	X	X					
Surgical intervention		X					

Assessment of Endpoints		X	X	X	X	X	X
Assessment of Safety		X	X	X	X	X	X
Sampling of Blood	X						

Table 2: Overview of trial visits

2.5 PLAN FOR FURTHER TREATMENT OF THE PATIENTS AFTER TERMINATION OF THE TRIAL

After termination of the trial, the trial subjects will be further treated and followed-up in regular intervals in the pancreatic consultation at the Department of General, Visceral, and Transplantation Surgery at the University of Heidelberg. In this consultation, all patients operated on the pancreas at the Department of General, Visceral, and Transplantation Surgery at the University of Heidelberg, are regularly assessed by specialists in the field of pancreatic surgery.

3. DATA MANAGEMENT

All protocol-required information collected during the trial must be entered by the investigator, or designated representative, in the CRF. The investigator, or designated representative, should complete the CRF pages as soon as possible after information is collected, preferably on the same day that a trial subject is seen for an examination, treatment, or any other trial procedure. Any outstanding entries must be completed immediately after the final examination. An explanation should be given for all missing data.

The completed CRF must be reviewed and signed by the investigator named in the trial protocol or by a designated sub-investigator. After keeping a copy at the trial center, the original CRF is sent to the SDGC (staff not included in trial conduct) for data entry.

In order to ensure that the database reproduces the CRFs correctly, the SDGC accomplishes a double entry of data. The completeness, validity and plausibility of data are examined by validating programs, which thereby generate queries. The investigator or the designated representatives are obliged to clarify or explain the queries. At the end of the trial the principal investigator will retain the originals of all CRFs.

The data will be managed and analyzed in accordance with the appropriate Standard Operating Procedures (SOPs) valid in the SDGC.

4. STATISTICAL PROCEDURES

4.1 SAMPLE SIZE CALCULATION

A total of 35 patients will be recruited to the trial and treated by the application of the 2-OCA surgical sealant to the surface of the pancreatic remnant after DP. This sample size is judged sufficient for the evaluation of safety and applicability in this setting. If the trial proves safety of the intervention, a multicenter efficacy RCT will be conducted consecutively.

4.2 ANALYSIS VARIABLES AND STATISTICAL METHODS

All patients treated with the trial intervention will be considered in the final analysis.

The empirical distribution of all endpoints will be calculated, including mean, standard deviation and quartiles in case of continuous variables and scores, and with absolute and relative frequencies in case of categorical data. 95% confidence intervals will be calculated. Whenever appropriate, statistical graphics will be used to visualize the findings.

Missing data will be minimized by consequent documentation and all other reasonable methods. No interpolation of missing data will be performed.

(Serious) adverse events will be summarized using descriptive statistics. The adverse events will be categorized as surgical, cardiovascular, pulmonary, urinary and other complications. Furthermore, it will be defined if events were device-related or not. Device-related and not device-related (serious) adverse events will be reported seperately. Proportions and frequencies of adverse events will be presented. Specific focus will be placed on potential device-related adverse events.

Because the BOND-Trial is designed as a proof-of-concept trial no formal analyses will be conducted. In case of safety and feasibility, the results will serve as a basis for sample size calculation (set into relation to the results from previous RCTs) for a succeding randomized controlled trial.

5. QUALITY ASSURANCE

5.1 CLINICAL DATA MONITORING

During the clinical trial, quality control and quality assurance will be ensured via monitoring, auditing and inspections by the competent authorities, if and when applicable. All investigators agree that the monitor can visit the center before, during and after completion of the study to ensure that the study is conducted, recorded and reported according to the study protocol, relevant standard operating procedures, requirements of GCP and the applicable regulatory requirements (e.g. DIN EN ISO 14155). In addition, audits can be conducted by the sponsor and/or by the competent authorities in accordance with ICH-GCP and DIN EN ISO 14155.[33] The aim of auditing is to assure that all results and conclusions written in the

final report can be drawn from the source data. This includes controlling of data filing and organisation of the study center as well as controlling of third parties and original documents.

5.2 Assessment of Safety

Definition and Documentation of Adverse Events

According to ISO 14155 an adverse event is any untoward medical occurrence, unintended disease or injury, or untoward clinical sign (including abnormal laboratory findings) in subjects, users or other persons, whether or not related to the investigational medical device.

Note:

This definition includes events related to the investigational medical device or the comparator.

This definition includes events related to the procedures involved.

For users or other persons, this definition is restricted to events related to investigational medical devices.

An AE may be:

- new symptom/ medical condition,
- new diagnosis,
- changes of laboratory parameters,
- intercurrent diseases and accidents,
- worsening of medical conditions/ diseases existing before clinical trial start,
- recurrence of disease,
- increase of frequency or intensity of episodical diseases.

A pre-existing disease or symptom will not be considered an adverse event unless there will be an untoward change in its intensity, frequency or quality. This change will be documented by an investigator.

Surgical procedures themselves are not AEs; they are therapeutic measures for conditions that require surgery. The condition for which the surgery is required may be an AE. All AEs (including SAEs) will be documented on an AE-form. AEs are classified as "non-serious" or "serious".

Serious Adverse Event (SAE)

According to the Ordinance on Medical Devices Vigilance (Medizinprodukte-Sicherheitsplanverordnung, MPSV), a serious adverse event (SAE) is defined as follows:

Any untoward event in a clinical trial which is subject to approval that indirectly or directly led, might lead or might have led to death or a serious deterioration in state of health of a patient, user or other person and which does not necessarily have a causal relationship to the medical device, with one of the following outcomes:

a. death

b. a serious deterioration in state of health of the patient which led to a

1) life- threatening illness or

2) permanent impairment of a body function or permanent damage to a body structure or

3) requirement of subject hospitalization or prolongation of existing hospitalization,

4) necessity of medical or surgical intervention to prevent permanent impairment of a body function or permanent damage to a body structure

c. foetal distress, foetal death or any congenital abnormality or birth defects

Note:

Planned hospitalization for a pre-existing condition, or a procedure required by the protocol, without serious deterioration in health, is not considered a serious adverse event.

Adverse Device Effect (ADE)

An adverse device effect is an adverse event related to the use of an investigational medical device.

Serious Adverse Device Effect (SADE)

A serious adverse device effect is a device effect that has resulted in any of the consequences characteristic of a serious adverse event.

Unanticipated Serious Adverse Device Effect (USADE)

An unanticipated serious adverse device effect (USADE) is a serious adverse device effect which by its nature, incidence, severity or outcome has not been identified in the current version of the risk analysis report, as contained in the investigator's brochure ("Handbuch des klinischen Prüfers").

In contrast, an anticipated serious adverse device effect (ASADE) is an effect which by its nature, incidence, severity or outcome has been identified in the risk analysis report.

All adverse events occuring during the period between the application of the medical device and the last follow-up visit must be documented on an "Adverse Event Form" in the CRF. All untoward medical occurrences appearing prior to the application of the medical device will be recorded as medical history.

<u>Classification of Intensity</u>

The intensity of a SAE will be classified as follows:

Mild:	Temporary event which is tolerated well by the subject
Moderate:	Event which results in discomfort for the subject and impairs his/her normal activity
Severe:	Event which results in substantial impairment of normal activities of subject

Classification of Outcome

The outcome of a SAE at the time of last contact with the subject is classified.

Ongoing: Signs and symptoms of the SAE still exist.

Recovered completely: All signs and symptoms of SAE have disappeared.

Recovered with sequelae: Acute signs and symptoms of SAE have disappeared, sequelae caused by the SAE still exist.

Death: The SAE has caused the death of the patient. If a subject has suffered from more than one SAE, only the outcome for the SAE directly responsible for death is classified as ‘death‘, the other SAEs are classified according to their specific outcome.

Unknown: The outcome is not known or is implausible and there is no possibility to complete or verify the information.

Classification of Causality

The causality will be classified in a binary order:

Reasonable possibility that the trial intervention caused the SAE? YES/NO

If causality is “not assessable” it will be classified as NO.

Classification of Countermeasures

The countermeasures will be documented according to the following rules:

None: No action taken

Drug treatment: Newly-prescribed medication or change in dose of a medication

Others: Other countermeasures, e.g. an operative procedure

Reporting of Serious Adverse Events

Immediate Reporting

According to § 3 (5) of the Ordinance on Medical Devices Vigilance (Medizinprodukte-Sicherheitsplanverordnung, MPSV), last updated on July 25th, 2014, all SAEs have to be reported immediately by the investigator to the sponsor.

The sponsor will report immediately to the national competent authority (Bundesinstitut für Arzneimittel und Medizinprodukte - BfArM) all SAEs concerning subjects, users or others, for which a causal relationship with the medical device under investigation, with applied diagnostic or therapeutic measures or other conditions of the clinical trial cannot be excluded. .All reports will be sent electronically using the latest version of the report form provided on the BfArM website

(http://www.bfarm.de/SharedDocs/Formulare/DE/Medizinprodukte/Meldeformular_Klinische-Pruef_SAE.pdf?__blob=publicationFile&v=4).

Periodic Reporting

All SAEs which do not fulfill the criteria for immediate reporting will be documented completely by the sponsor and summarized. They will be reported quarterly to the BfArM or upon request using the SAE reporting table form provided on the EC website (http://ec.europa.eu/health/medical-devices/documents/guidelines/index_en.htm).

Actions of Sponsor

The sponsor has to assure, that every SAE will be reported by all investigators participating in this trial. Serious adverse events have to be reported by the attending physician to the principal investigator within 24 hours after the incident. The initial report must be as complete as possible including details of the current illness and (serious) adverse event and an assessment of the causal relationship between the event and the trial treatment.

The sponsor is responsible for the classification of serious adverse events and ongoing safety evaluation of the clinical investigation and shall review the investigator's assessment of all serious adverse events and determine and document in writing their seriousness and relationship to the investigational device; in case of disagreement between the sponsor and the investigator(s), the sponsor shall communicate both opinions.

The sponsor should assess whether corrective or preventive action is required. If the sponsor himself takes independently corrective actions to ensure the safety of subjects, users or others, the national competent authority and in this case also the ethics committee have to be informed immediately.

Reporting of serious adverse events to:

PD Dr. Markus Diener

Studienzentrum der Deutschen Gesellschaft für Chirurgie

Im Neuenheimer Feld 110

69120 Heidelberg

Pursuant to the German Medical Device Law (MPG) and the Ordinance on Medical Devices Vigilance (MPSV) the competent authority (BfArM) will be informed of all SAEs during the trial. The BfArM and the IEC (if necessary) will be informed in case the risk/benefit assessment changes or any other new and significant hazards for subjects' safety or welfare occur.

The competent authority must be informed within 90 days upon completion about the end of the trial. It will be provided with a summary of trial results within one year after the end of clinical phase (LPO) (see §23a MPG).

Emergency treatment

During and following a subject's participation in the trial, the investigator must ensure that adequate medical care is provided. The subject must receive adequate treatment in any clinical situation including emergencies and outcome of the patient must be controlled.

5.2.1 Data safety and monitoring board (DSMB)

In case of any irregularities for example concerning the frequency or type of SAE reported the principal investigator will inform the members of the independent Data Safety Monitoring Board (DSMB) without delay. At least once every 3 months, the DSMB will receive a written safety report. After reception of the semestral report, the members of the DSMB will discuss the safety report in a telephone conference. The members of the DSMB then report the result of the benefit/risk assessment to the steering committee and will give appropriate recommendations concerning the continuation of the trial.

The Members of the DSMB are stated above (see "ROLES AND RESPONSIBILITIES").

5.3 STEERING COMMITTEE

A steering committee will be established (see Roles and Responsibilities). The steering committee will supervise the conduct of the trial and will issue recommendations for early termination, modifications or continuation of the trial, if necessary. Regular meetings of the steering committee will be held during the whole course of the trial.

5.4 RECORD RETENTION AND DIRECT ACCESS TO SOURCE DATA/DOCUMENTS

The SDGC will maintain the records of the study consisting of all correspondence with the relevant authorities and ethics committee, study protocol with any amendments, investigator's brochure, the patients' signed informed consent forms, investigational device accountability records, and individual subject records. All source data and relevant

documents will be kept for at least 2 years after this clinical trial is terminated. Thereafter, documents will be archived for at least 10 years after termination of the trial as required by the MPKPV. Other regulations for the storage of medical records stay unaffected by this procedure.

According to the MPKPV the investigators must provide direct access to source data for trial related monitoring, audits and regulatory inspections. Each subject has consented – via written informed consent – to direct access to his/her original medical recrds for trial-related monitoring, audit and regulatory inspection.

All data and documents will be made available if requested by the relevant authorities.

6. DEVICE ACCOUNTABILITY

The surgical sealant will be provided to the study site by the SDGC after all required regulatory documentation has been received. A label stating "Exclusively for clinical investigation" will be applied and the device will be stored in a locked area at the appropriate storage conditions. Access will be limited to designated study staff. A device accountability log will be provided. Every disposition of the device will be documented referring to the individual study subject (including units used per patient).

In the device accountability log, potential product deficiencies will be documented. In case of SAEs a potential connection between device deficiencies and SAE can be assessed by review of the documentation.

7. ETHICAL AND LEGAL ASPECTS

As described in the sections 1.1 and 2.2.3 above, 2-OCA is a surgical glue which promises benefit in the prevention of POPF. From the available results in the literature, 2-OCA seems to be safe and has already been approved for the intracorporal use.

Distal pancreatic resection is a standard operative technique for pancreatic lesions in the body or tail of the gland, which is used on a day-to-day basis at the surgical department of the University Hospital Heidelberg.

Nevertheless, we will closely monitor the occurrence of all (serious) adverse events.

Before the acquisition of any data, patients will be informed about the trial and the data handling and have to provide written informed consent. All data will be handled according to the German Data Protection Law. Data will be pseudonymized and access to data will only be given to authorized persons, e.g. monitors and the competent authorities (BfArM).

The study will be conducted in accordance with the Declaration of Helsinki the international principles of Good Clinical Practice (ICH-GCP) and all applicable laws, e.g. the German Medical Device Law (Medizinproduktegesetz = MPG), the European Medical Device Directive 93/42/EWG (amended by 2007/47/EG), and hamonised norms (DIN EN ISO 13485, DIN EN ISO 10993, DIN ISO 11135, EN 550, DIN EN ISO 14155, DIN EN ISO 14971).

A patient's insurance will be provided in accordance with the German Medical Device Law.

The insurance was taken out at

HDI-Gerling Versicherungs AG, Postfach 510369, 30633 Hannover

MPG insurance number: 57 010310 03018

Any impairment of health which might occur in consequence of trial participation must be notified to the insurance company. The patient is responsible for notification. The insured person will be agreed to all appropriate measures serving for clarification of the cause and the extent of damage as well as the reduction of damage. During the conduct of the trial, the patient must not undergo other clinical treatment except for cases of emergency. The patient is bound to inform the investigator immediately about any adverse events and additionally drugs taken. The terms and conditions of the insurance should be delivered to the patient.

The insurance company has to be informed about all amendments that could affect patients' safety.

7.1 PREMATURE TERMINATION OF THE TRIAL

The trial may be closed prematurely by the principal investigator in consultation with the steering committee and the responsible biometrician.

If the termination of the trial becomes necessary, the steering committee of the trial will discuss this issue with the independent Data Safety Monitoring Board (DSMB). Reasons that may necessitate a termination of the trial include the following:

- The incidence or severity of serious adverse events/morbidity in this trial indicates a potential health hazard caused by the trial intervention.
- It appears that patients' enrolment is unsatisfactory with respect to quality and/or quantity or data recording is severely inaccurate and/or incomplete.
- External evidence demanding a termination of the trial.

The independent ethics committee (IEC) and the corresponding authorities (BfArM) will then be informed.

7.2 PROTOCOL APPROVAL AND AMENDMENTS

Before the start of the trial, the trial protocol, informed consent document, and any other appropriate documents will be submitted to the independent EC and the corresponding authorities (BfArM). Also all amendments will be submitted to the ECs and the corresponding authorities. Formal approval by the EC should preferably mention the title of the trial, the trial code, the trial site, and any other documents reviewed. Changes will not be implemented until approval of the IEC and the BfArM have been given.

7.3 RESPONSIBILITIES OF INVESTIGATOR

The principal investigator should ensure that all persons assisting in the trial are adequately informed about the protocol, any amendments to the protocol, the investigator's brochure, the medical device, the trial treatments, and their trial-related duties and functions.
The investigator should maintain a list of sub-investigators and other appropriately qualified persons to whom he or she has delegated significant trial-related duties.

8. AGREEMENTS

8.1 FINAL REPORT

In accordance with §23a of the MPG the final report of the trial will be submitted to the BfArM within 12 months after the end of the clinical phase (LPO).

8.2 FINANCING OF THE TRIAL

The trial was partially funded by a grant of the Heidelberger Stiftung Chirurgie, Im Neuenheimer Feld 110, 69120 Heidelberg.

8.3 DISSEMINATION

The trial will be registered in an international trial registry (e.g. www.clinicaltrials.gov). Simultaneously to trial start, the protocol will be published.

Results of the BOND trial will be presented to the national and international surgical community at (inter-)national surgical conferences. Furthermore, the final report will be published in an international peer-reviewed journal.

After completion of the trial and if the trial intervention proves to be safe, a multicenter RCT will be set up for the evaluation of efficacy.

The Department of General, Visceral, and Transplantation Surgery of the University of Heidelberg in its role as the European Pancreas Center has an exemplary function for the treatment of pancreatic diseases worldwide.

Besides that, the SDGC promotes evidence-based surgery and evidence-based patient information on a national and international level, via publications, scientific presentations, internet presence, the German Surgical Research Network, CHIR-Net, and with training programs and educational courses for surgeons.

8.4 TRANSLATIONAL RESEARCH

The basic and clinical research aims to identify parameters which influence the clinical outcome and may be useful for prognostic or therapeutical decision making in future with main focus on POPF. Serum parameters (20 ml blood) are investigated in a standardised fashion. A proteomic screening is planned for potential factors influencing the occurrence of POPF. The alterations are analysed in detail and assessed for their clinical implications. The main focus will be an explorative evaluation of potential risk factors for POPF, which can be assessed preoperatively. So in the future, potential individual treatment decisions can be made on the preoperative assessment of these factors.

Potential markers include the type I/III-collagen ratio, MMPs, TIMPs etc., which are related to cell adhesions and the integrity of connective tissues. All investigations will be performed centralised and all data stored in a separate database. This will be linked to the clinical database to answer the above mentioned questions.

9. REFERENCES

1. Bassi, C., et al., *Postoperative pancreatic fistula: an international study group (ISGPF) definition.* Surgery, 2005. **138**(1): p. 8-13.
2. Wente, M.N., et al., *Delayed gastric emptying (DGE) after pancreatic surgery: a suggested definition by the International Study Group of Pancreatic Surgery (ISGPS).* Surgery, 2007. **142**(5): p. 761-8.
3. Wente, M.N., et al., *Postpancreatectomy hemorrhage (PPH): an International Study Group of Pancreatic Surgery (ISGPS) definition.* Surgery, 2007. **142**(1): p. 20-5.
4. McCulloch, P., et al., *No surgical innovation without evaluation: the IDEAL recommendations.*Lancet, 2009. **374**(9695): p. 1105-12.
5. *Krebs in Deutschland 2007/2008.* Robert Koch-Institut und die Gesellschaft der epidemiologischen Krebsregister in Deutschland e.V.: Berlin, 2012.
6. *Fallpauschalenbezogene Krankenhausstatistik (DRG-Statistik) Operationen und Prozeduren der vollstationären Patientinnen und Patienten in Krankenhäusern - Ausführliche Darstellung - 2011.* Statistisches Bundesamt: Wiesbaden, 2012.
7. Buchler, M.W., et al., *Changes in morbidity after pancreatic resection: toward the end of completion pancreatectomy.* Arch Surg, 2003. **138**(12): p. 1310-4; discussion 1315.
8. Hackert, T., et al., *Enucleation in pancreatic surgery: indications, technique, and outcome compared to standard pancreatic resections.* Langenbecks Arch Surg, 2011. **396**(8): p. 1197-203.
9. McPhee, J.T., et al., *Perioperative mortality for pancreatectomy: a national perspective.* Ann Surg, 2007. **246**(2): p. 246-53.
10. Melloul, E., et al., *Poor level of agreement on the management of postoperative pancreatic fistula: results of an international survey.* HPB (Oxford), 2013. **15**(4): p. 307-14.

11. Lermite, E., et al., *Complications after pancreatic resection: diagnosis, prevention and management.* Clin Res Hepatol Gastroenterol, 2013. **37**(3): p. 230-9.
12. Diener, M.K., et al., *Efficacy of stapler versus hand-sewn closure after distal pancreatectomy (DISPACT): a randomised, controlled multicentre trial.* Lancet, 2011. **377**(9776): p. 1514-22.
13. Gebauer, F., et al., *Options and limitations in applying the fistula classification by the International Study Group for Pancreatic Fistula.* Ann Surg, 2012. **256**(1): p. 130-8.
14. Frymerman, A.S., et al., *Impact of postoperative pancreatic fistula on surgical outcome--the need for a classification-driven risk management.* J Gastrointest Surg, 2010. **14**(4): p. 711-8.
15. Hackert, T. and M.W. Buchler, *Remnant closure after distal pancreatectomy: current state and future perspectives.* Surgeon, 2012. **10**(2): p. 95-101.
16. Carter, T.I., et al., *A dual-institution randomized controlled trial of remnant closure after distal pancreatectomy: does the addition of a falciform patch and fibrin glue improve outcomes?* J Gastrointest Surg, 2013. **17**(1): p. 102-9.
17. Montorsi, M., et al., *Efficacy of an absorbable fibrin sealant patch (TachoSil) after distal pancreatectomy: a multicenter, randomized, controlled trial.* Ann Surg, 2012. **256**(5): p. 853-9; discussion 859-60.
18. Orci, L.A., et al., *Systematic review and meta-analysis of fibrin sealants for patients undergoing pancreatic resection.* HPB (Oxford), 2013.
19. Satoi, S., et al., *Reinforcement of pancreticojejunostomy using polyglycolic acid mesh and fibrin glue sealant.* Pancreas, 2011. **40**(1): p. 16-20.
20. Eaglstein, W.H. and T. Sullivan, *Cyanoacrylates for skin closure.* Dermatol Clin, 2005. **23**(2): p. 193-8.
21. Barakat, O., C.F. Ozaki, and R.P. Wood, *Topically applied 2-octyl cyanoacrylate (Dermabond) for prevention of postoperative pancreatic fistula after pancreaticoduodenectomy.* J Gastrointest Surg, 2012. **16**(8): p. 1499-507.
22. Diener, M.K., et al., *DISPACT trial: a randomized controlled trial to compare two different surgical techniques of DIStal PAnCreaTectomy - study rationale and design.* Clin Trials, 2008. **5**(5): p. 534-45.
23. Schenk, W.G., 3rd, et al., *Absorbable cyanoacrylate as a vascular hemostatic sealant: a preliminary trial.* Am Surg, 2005. **71**(8): p. 658-61.
24. Lumsden, A.B., E.R. Heyman, and G. Closure Medical Surgical Sealant Study, *Prospective randomized study evaluating an absorbable cyanoacrylate for use in vascular reconstructions.* J Vasc Surg, 2006. **44**(5): p. 1002-1009; discussion 1009.
25. Brunkwall, J., et al., *A single arm, prospective study of an absorbable cyanoacrylate surgical sealant for use in vascular reconstructions as an adjunct to conventional techniques to achieve haemostasis.* J Cardiovasc Surg (Torino), 2007. **48**(4): p. 471-6.
26. Carnero-Alcazar, M., et al., *Short-term and mid-term follow-up of sutureless surgery for postinfarction subacute free wall rupture.* Interact Cardiovasc Thorac Surg, 2009. **8**(6): p. 619-23.
27. Carr, J.A., *The intracorporeal use of 2-octyl cyanoacrylate resin to control air leaks after lung resection.* Eur J Cardiothorac Surg, 2011. **39**(4): p. 579-83.
28. Aziz, O., et al., *Novel applications of Dermabond (2-octyl -cyanoacrylate) in cardiothoracic surgery.* Surg Technol Int, 2007. **16**: p. 46-51.
29. Binmoeller, K.F., et al., *EUS-guided transesophageal treatment of gastric fundal varices with combined coiling and cyanoacrylate glue injection (with videos).* Gastrointest Endosc, 2011. **74**(5): p. 1019-25.
30. Rengstorff, D.S. and K.F. Binmoeller, *A pilot study of 2-octyl cyanoacrylate injection for treatment of gastric fundal varices in humans.* Gastrointest Endosc, 2004. **59**(4): p. 553-8.

31. Tian, X., et al., *Modified percutaneous transhepatic variceal embolization with 2-octylcyanoacrylate for bleeding gastric varices: long-term follow-up outcomes.* AJR Am J Roentgenol, 2011. **197**(2): p. 502-9.
32. Wang, J., et al., *Comparison of modified percutaneous transhepatic variceal embolization and endoscopic cyanoacrylate injection for gastric variceal rebleeding.* World J Gastroenterol, 2013. **19**(5): p. 706-14.
33. International Conference on Harmonisation of technical requirements for registration of pharmaceuticals for human, u., *ICH harmonized tripartite guideline: Guideline for Good Clinical Practice.* J Postgrad Med, 2001. **47**(1): p. 45-50.

10. DECLARATION OF INVESTIGATOR

I have read the above trial protocol and I confirm that it contains all information to accordingly conduct the clinical trial. I pledge to conduct the clinical trial according to the protocol.
I will enroll the first subject only after all ethical and regulatory requirements are fulfilled. I pledge to obtain written consent for trial participation from all subjects.

I know the requirements for accurate notification of serious adverse events and I pledge to document and notify such events as described in the protocol.

I pledge to retain all trial-related documents and source data as described. I will provide a curriculumvitae (CV) before trial start.

Name (block letters): ______________________________

Function: Investigator

Trial Center (address): ______________________________

____

____

Date: ______________________________

Signature: ______________________________

APPENDICES

APPENDIX I: OMNEX INSTRUCTIONS FOR USE (EFFECTIVE JULY 2014)

ETHICON™

omnex™

SURGICAL SEALANT

KIRURGISK VÆVSLIM

CHIRURGISCHE WEEFSELLIJM

KIRURGINEN TIIVISTEAINE

COLLE CHIRURGICALE

CHIRURGISCHER KLEBER

ΧΕΙΡΟΥΡΓΙΚΗ ΣΤΕΓΑΝΩΤΙΚΗ ΟΥΣΙΑ

SIGILLANTE CHIRURGICO

KIRURGISK TETNINGSMASSE

VEDANTE CIRÚRGICO

SELLANTE QUIRÚRGICO

KIRURGISKT FÖRSEGLINGSMEDEL

CERRAHİ İZOLASYON MADDESİ

ETHICON™

ETHICON, LLC
San Lorenzo, Puerto Rico 00754

© Ethicon, Inc. 2009

CE 0086

ENGLISH

ETHICON™

omnex™

SURGICAL SEALANT

DESCRIPTION

ETHICON™ OMNEX™ Surgical Sealant is a synthetic tissue sealant consisting of a blend of two monomers, 2-octyl cyanoacrylate (2-OCA) and butyl lactoyl cyanoacylate (BLCA). The liquid formulation is contained in a crushable glass ampoule, which is housed in a molded unit. The formulation is passed through a porous disc containing an initiator, mixed in a chamber, and delivered through a cannula. Following standard closure techniques, ETHICON™ OMNEX™ Surgical Sealant is applied to the anastomotic closure line, including suture, staple, and clip holes. The sealant polymerizes to form a film adherent to the tissue and/or synthetic material and creates a flexible physical seal, independent of the body's clotting mechanism. The formation of this flexible physical seal prevents leakage of blood. ETHICON™ OMNEX™ Surgical Sealant is designed to provide a strong physical seal that remains in place beyond the time required for natural healing, and eventually degrades over time, breaking down into smaller absorbable fragments.

The applicator is designed to provide consistent mixing of the components prior to application. The sterile device is provided as a packaged single-use applicator and stored at room temperature.

INDICATIONS FOR USE

ETHICON™ OMNEX™ Surgical Sealant is indicated for use in vascular reconstructions to achieve adjunctive hemostasis by mechanically sealing areas of leakage.

CONTRAINDICATIONS

- Do not use on patients with known hypersensitivity to cyanoacrylate or formaldehyde.
- Not for intravascular use.

WARNINGS

- ETHICON™ OMNEX™ Surgical Sealant is intended for use as an adjunctive sealant and is not to be used in place of sutures, staples, or mechanical closure.
- Biological fluids and other residual moisture must be removed from the entire circumference of the anastomosis prior to application and maintained for two (2) minutes post application. Proper clamping, clipping or ligation prevents blood seepage during application.
- Very small amounts of the product are required to create an effective seal. ETHICON™ OMNEX™ Surgical Sealant should be applied to the dry anastomotic closure lines by expressing a partial drop and evenly spreading into a thin film.
- ETHICON™ OMNEX™ Surgical Sealant is a fast setting sealant capable of adhering to body tissues and many other materials, such as latex gloves, and surgical instruments. Take necessary precautions to avoid contact with unintended surfaces.
- The cannula contains a stainless steel wire, which allows the user to bend the cannula and position it for sealant application in more difficult to reach areas.
- The cannula tip should not be cut or trimmed due to the risk of exposing the "memory" wire.

PRECAUTIONS

- Excessive pressure of the applicator's cannula tip against the vessel/graft edges or surrounding tissue can result in forcing the vessel/graft edges apart and allowing the sealant into the vessel. ETHICON™ OMNEX™ Surgical Sealant within the vessel could delay wound healing and/or result in local or embolic vascular obstruction.
- Avoid applying ETHICON™ OMNEX™ Surgical Sealant to anastomoses or suture lines when the lumen of the vessel is under negative pressure or suction to avoid unpolymerized product from being drawn into the vessel lumen. This concern is most relevant during cardiac procedures requiring closure and de-airing of the aorta.
- ETHICON™ OMNEX™ Surgical Sealant has not been clinically evaluated for use on coronary artery anastomoses or on the myocardium.
- ETHICON™ OMNEX™ Surgical Sealant has not been clinically evaluated for use in pediatrics.
- Peeling ETHICON™ OMNEX™ Surgical Sealant after it has polymerized from application sites, both intended and unintended sites, could result in tissue damage.

ADVERSE REACTIONS

In a prospective, randomized, controlled, multi-center trial, one hundred fifty-one (151) patients were treated with ETHICON™ OMNEX™ Surgical Sealant or the control (oxidized regenerated cellulose). Table 1 provides a summary of vascular and bleeding complication adverse events reported for the ETHICON™ OMNEX™ Surgical Sealant treated and control treated patients.

2

Table 1: Vascular or Bleeding Complications Adverse Events

	ETHICON™ OMNEX™ Surgical Sealant (N=101) n (%)	Control (N=50) n (%)	P-Value
**Number of patients with at least 1 Vascular or Bleeding Complication**	23 (22.8%)	20 (40.0%)	0.035
**Bleeding Complications**			
Bleeding, procedure	2 (2.0%)	0 (0%)	1.0
Bleeding, post procedure	2 (2.0%)	1 (2.0%)	1.0
Hematoma	2 (2.0%)	6 (12.0%)	0.016
Coagulopathy	1 (1.0%)	0 (0%)	1.0
**Vascular Complications**			
Occlusion of Graft/Vessel	12 (11.9%)	8 (16.0%)	0.48
Edema	5 (5.0%)	4 (8.0%)	0.48
Thrombosis	5 (5.0%)	3 (6.0%)	1.0

Results show that the number of patients with at least one vascular or bleeding complication was greater in the control than ETHICON™ OMNEX™ Surgical Sealant, and the difference was statistically significant (P-Value, 0.035).

Table 2 shows all other adverse events reported by three or more patients treated with ETHICON™ OMNEX™ Surgical Sealant or control.

Table 2: Other Adverse Event[1] Complications Reported by Three or More Patients Treated

	ETHICON™ OMNEX™ Surgical Sealant (N=101) n (%)	Control (N=50) n (%)	P-Value
Infection[2]	10 (9.9%)	8 (16.0%)	0.29
Pain	9 (8.9%)	5 (10.0%)	1.0
Erythema	9 (8.9%)	1 (2.0%)	0.17
Wound Infection[3]	8 (7.9%)	2 (4.0%)	0.50
Dehiscence	5 (5.0%)	0 (0%)	0.17
Renal Failure	4 (4.0%)	1 (2.0%)	1.0
Lymphocele/Lymph Fistula	3 (3.0%)	0 (0%)	0.55

1. All adverse events other than vascular or bleeding complications reported
2. Infection was defined as non-wound infections
3. Wound infection was defined as surgical incision site infections reported

The results are similar between the two treatment groups and are representative of events expected from patients undergoing vascular surgery for vascular access or occlusive vascular disease with the exception of hematoma (control 12% and ETHICON™ OMNEX™ Surgical Sealant 2%; P-Value, 0.016). There were no unanticipated adverse device effects (UADE) in this investigation.

CLINICAL STUDIES

United States & European Union Multi-Center Pivotal Study

Study Design and Objectives:

A prospective, randomized, controlled, multi-center trial was conducted to evaluate the safety and effectiveness of ETHICON™ OMNEX™ Surgical Sealant versus control, oxidized regenerated cellulose, to seal anastomotic suture lines in patients undergoing vascular reconstruction procedures receiving an ePTFE graft. One hundred fifty-one patients (151) were enrolled at 13 centers. The objective of the study was to collect clinical data concerning the safety and effectiveness of ETHICON™ OMNEX™ Surgical Sealant for use as an anastomotic sealant to provide hemostasis and shows superiority to the control, which is considered the standard of care.

Of the 151 patients, 101 patients were treated with ETHICON™ OMNEX™ Surgical Sealant and 50 patients were treated with the control.

3

Table 3: Patient Demographics by Age, Gender, and Surgical Procedure

	ETHICON™ OMNEX™ Surgical Sealant (N = 101)	Control (N = 50)
**Age**		
Mean ± SD	60.8 ± 14.3	61.4 ± 13.9
Median	62.0	61.0
Range	21 – 96	29 – 90
**Gender**		
Males: n (%)	66 (65.4%)	28 (56.0%)
Females: n (%)	35 (34.6%)	22 (44.0%)
**Procedure**		
Femoral Bypass: n (%)	46 (45.5%)	23 (46.0%)
AV Access for Hemodialysis: n (%)	55 (54.5%)	27 (54.0%)

Table 4: Patient Accountability

	ETHICON™ OMNEX™ Surgical Sealant	Control
**Total Number of Patients Treated:**	101	50
Number of Patients with 1 Site Treated:	57	27
Number of Patients with 2 Sites Treated:	40	21
Number of Patients with 3 Sites Treated:	4	2
**Total Number of Sites Treated:**	149	73

Primary Endpoint:

The primary effectiveness outcome parameter measured was time to hemostasis for patients treated with ETHICON™ OMNEX™ Surgical Sealant to that of patients treated with the control. For femoral bypass patients with more than one anastomotic site treated, the site with the longest time to hemostasis was used in the analysis.

Table 5: Time to Hemostasis (sec)[1] Summary – All Patients

	ETHICON™ OMNEX™ Surgical Sealant	Control
Mean Time to Hemostasis[2,3]	119.3	403.8

1. The anastomotic site with the longest time to hemostasis was used for femoral bypass patients with multiple sites treated. All times > 10 minutes were replaced by 10 minutes.
2. Adjusted for study center and type of procedure
3. Test of hypothesis that ETHICON™ OMNEX™ Surgical Sealant mean is no more than one minute longer than that of the control; test of non-inferiority P-Value is < 0.001; Test of superiority P-Value is < 0.001

Multiple analyses were conducted to evaluate the effectiveness data by procedural type and by patient. These analyses demonstrated that all study objectives were met. The results of the study showed that patients who received ETHICON™ OMNEX™ Surgical Sealant had a statistically significant faster time to hemostasis than that of the control ($p < 0.001$).

Secondary Endpoints:

The secondary effectiveness endpoints were: (1) the number of patients who achieved IMMEDIATE hemostasis (at time 0), and within 1, 5, and 10 minutes and (2) the number of patients who required additional adjunctive agents in order to achieve hemostasis.

4

Table 6: Time to Hemostasis[1] by Minute Intervals for All Procedures – All Patients

Interval[2]	ETHICON™ OMNEX™ Surgical Sealant	Control
0 (immediate)	55 (54.5%)	5 (10.0%)
0-1 minute	61 (60.4%)	7 (14.0%)
0-5 minutes	89 (88.1%)	16 (32.0%)
0-10 minutes	94 (93.1%)	29 (58.0%)
> 10 minutes	7 (6.9%)	21 (42.0%)

1. The anastomotic site with the longest time to hemostasis was used for femoral bypass patients with multiple sites treated. All times > 10 minutes were replaced by 10 minutes
2. Adjusted for study center and type of procedure; P-Values for all time intervals is < 0.001

As with the primary variable, femoral bypass patients with more than one anastomotic site treated had the site with the longest time to hemostasis used. These data were analyzed by the Cochran-Mantel-Haenszel procedure, stratified by the cross classification of the study center and procedure. The proportion of patients achieving hemostasis within each of the intervals was statistically significantly greater for patients treated with ETHICON™ OMNEX™ Surgical Sealant than for patient treated with the control ($p < 0.001$).

Table 7: Use of Additional Adjunctive Agents to Achieve Hemostasis – All Patients

	ETHICON™ OMNEX™ Surgical Sealant n (%)	Control n (%)
At least one additional agent required[1]	31 (30.7%)	22 (44.0%)
One additional unit of assigned treatment	11 (10.9%)	15 (30.0%)
Stitches	7 (6.9%)	7 (14.0%)
Pledgets	0 (0%)	0 (0%)
Protamine	7 (6.9%)	8 (16.0%)
Other	12 (11.9%)	3 (6.0%)

1. Total number of individual agents may exceed the number of patients who had at least one agent used because patients may have had multiple agents used and because multiple anastomotic sites were treated in femoral bypass patients

Use of additional adjunctive agents was analyzed by the Cochran-Mantel-Haenszel procedure, stratified by the cross classification of study center and procedure. Femoral bypass patients who required an additional agent for any anastomotic site were classified as having required use of the additional agent. A greater proportion of patients in the control group (44.0%) required at least one additional agent than patients in the ETHICON™ OMNEX™ Surgical Sealant group (31%), although the difference did not achieve statistical significance (P-Value 0.08).

European Multi-center Study

A prospective, non-randomized, multi-center trial was conducted in Germany to evaluate the safety and effectiveness of ETHICON™ OMNEX™ Surgical Sealant to seal anastomotic suture lines in patients undergoing vascular reconstruction procedures using various types of graft materials. One hundred five patients (105) were enrolled at five (5) study centers.

The primary effectiveness endpoint was time to hemostasis. Overall, immediate hemostasis (at time 0) was achieved in 71% of the 158 application sites from 105 treated patients. Hemostasis was achieved in 94% of application sites within one minute; in the remaining 6% of application sites, hemostasis was achieved within eight minutes. Overall, mean time to hemostasis by anastomotic site was 23.2 seconds with a 95% confidence interval of 11.0 to 35.3 seconds.

5

Table 8: Time to Hemostasis

		AV Access Procedure	Bypass & AAA	Endarterectomy & Patch	Total
By Patient	# of Patients	7	75	23	105
	Mean (sec)	4.6 ± 8.5	39.6 ± 104.6	23.1 ± 55.9	33.7 ± 92.5
	Median (sec)	0.0	0.0	5.0	0.0
	Range (sec)	0 – 22	0 – 480	0 – 266	0 - 480
By Anastomoses	# of Anastomotic Sites	10	124[1]	24	158
	Mean (sec)	3.2 ± 7.3	25.0 ± 83.7	22.1 ± 54.9	23.2 ± 77.2
	Median (sec)	0.0	0.0	2.5	0.0
	Range (sec)	0 – 22	0 – 480	0 – 266	0 - 480

1. Time to Hemostasis was not captured at one anastomotic site

Secondary effectiveness variables included the number of anastomotic sites receiving each type of graft material and the number of patients who achieved hemostasis within 0 (immediate), 1, 5, and 10 minutes.

Table 9: Graft Material Used

Graft Material	AV Access Procedure	Bypass & AAA	Endarterectomy & Patch	Total[1]
PTFE	2 (20.0%)	51 (41.1%)	1 (4.2%)	54 (34.2%)
Dacron	0 (0.0%)	31 (25.0%)	18 (75.0%)	49 (31.0%)
Autologous	7 (70.0%)	42 (33.9%)	4 (16.7%)	53 (33.5%)
Other	1 (10.0%)	0 (0.0%)	1 (4.2%)	2 (1.3%)

1. Number of anastomotic sites treated

Table 10: Time to Hemostasis by Graft type

Graft Type	# of Anastomotic Sites	Time Interval			
		0 (immediate)	0-1 minute	0-5 minutes	0-10 minutes
PTFE	54	35 (64.8%)	46 (85.2%)	49 (90.7%)	54 (100.0%)
Dacron[1]	48	32 (66.7%)	47 (97.9%)	48 (100.0%)	48 (100.0%)
Autologous	53	43 (81.1%)	52 (98.1%)	53 (100.0%)	53 (100.0%)
Other	2	2 (100.0%)	2 (100.0%)	2 (100.0%)	2 (100.0%)

1. Time to Hemostasis was not captured at one anastomotic site in this group

There were no significant adverse events related to the product use reported in the single arm European trial. The events reported were typical of patients with clinical conditions related to vascular surgeries.

DIRECTIONS FOR USE

Prior to application of ETHICON™ OMNEX™ Surgical Sealant, the vessel segment that is being treated should be clamped and depressurized. The anastomotic site should be mechanically closed with sutures or staples.

APPLICATOR PREPARATION:

1. Remove the ETHICON™ OMNEX™ Surgical Sealant applicator from the sterile pouch and hold the applicator with the cannula tip pointing downward.
2. Apply pressure to the applicator lever to crush the inner glass ampoule. Then, release pressure on the applicator lever.
3. Squeeze the applicator lever again allowing the liquid to completely express into the cannula cover (i.e. mixing chamber). Avoid creating excessive foam or bubbles by expressing the liquid slowly into the mixing chamber. Then, release pressure on the applicator allowing the liquid to draw back into the applicator unit. **Repeat this mixing step two (2) additional times. Thorough mixing is essential for optimal performance.**
4. Remove and discard the cannula cover. The applicator is now ready for use; the target surgical field should be prepared for application.

6

SITE PREPARATION AND APPLICATION:

1. Prepare the anastomotic site to be treated by patting dry with dry, sterile gauze or a sterile sponge. For proper adherence, ETHICON™ OMNEX™ Surgical Sealant must have direct contact with the tissue or graft material.
2. **Very small amounts of sealant are required to create an effective seal.** Apply ETHICON™ OMNEX™ Surgical Sealant to the **dry anastomotic closure line of the clamped/depressurized vessel.** Squeeze partial drops and spread with the applicator tip on the anastomotic surface to create a thin film.
3. Ensure complete application over the anastomotic closure lines including all suture bites, needle holes, staple holes, and clip holes. ETHICON™ OMNEX™ Surgical Sealant forms a flexible polymeric seal at the tissue surface when completely set.
4. Allow two minutes (120 seconds) to pass before removing clamps to assure complete polymerization of ETHICON™ OMNEX™ Surgical Sealant. Verify that ETHICON™ OMNEX™ Surgical Sealant has set by gently touching the seal with the tip of the applicator. Full polymerization is confirmed when the thin film of sealant is no longer tacky.
5. Inspect and verify that the seal is effective. In the event excessive bleeding is observed after clamp removal, re-clamp, pat dry and reapply ETHICON™ OMNEX™ Surgical Sealant as previously indicated, or use other adjunctive therapies.

HOW SUPPLIED

Discard any unused material following completion of the medical procedure.

STORAGE

25°C 77°F max Do not use after expiry date.

STERILITY

STERILE EO STERILE

REPORTING: Direct all correspondence to your local distributor.

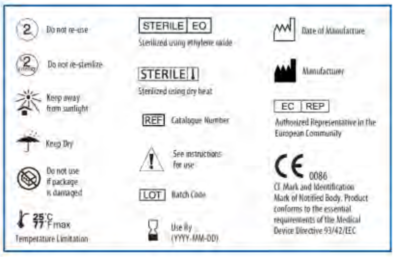

7

Distributors • Distributører • Distributeurs
Tukkumyyjät • Distributeurs • Vertriebe
Distributori • Distribuidores • Distribuidores
Distributörer • Διανομείς

Johnson & Johnson Wound Management
ETHICON S.A.S.
1Rue Camille Desmoulins
TSA 81002
92787 Issy Les Moulineaux Cedex 9
France

Johnson & Johnson Wound Management
Via del Mare 56
00040 Pomezia
Roma
Italia

CH: Johnson & Johnson AG
Rotzenbuehlstrasse 55
8957 Spreitenbach

Johnson & Johnson Medical Ltd.
Coronation Road
Ascot, Berks
SL5 9EY
United Kingdom

Johnson & Johnson Wound Management
Oststrasse 1
22844 Norderstedt
Germany

Authorized Representative • Autoriseret repræsentant
Erkende vertegenwoordiger • Valtuutettu edustaja
Représentant autorisé • Autorisierte Vertreter
Rappresentante autorizzato • Representante autorizado
Representante autorizado • Auktoriserad representant
Εξουσιοδοτημένος Αντιπρόσωπος

ETHICON, division of Johnson & Johnson Medical Limited
PO Box 1988
Simpson Parkway
Kirkton Campus
Livingston
EH54 0AB
Scotland

PM-7-2071-001 B

Johnson & Johnson
Wound Management
A division of ETHICON, INC.
Somerville, New Jersey 08876-0151

Manufactured for
Ethicon, Inc.
by Closure Medical Corp.
Raleigh, North Carolina 27616

APPENDIX II: FDA'S SUMMARY OF SAFETY AND EFFECTIVENESS DATA

SUMMARY OF SAFETY AND EFFECTIVENESS DATA (SSED)

I. GENERAL INFORMATION

Device Generic Name:	Polymerizing Sealant
Device Trade Name:	Ethicon™ OMNEX™ Surgical Sealant
Applicant Name and Address:	Ethicon, Inc. (Raleigh)<br>5250 Greens Dairy Road<br>Raleigh, NC 27616<br>United States of America
Date(s) of Panel Recommendation:	None
PMA Application Number:	P060029
Date of FDA Notice of Approval:	June 3, 2010
Expedited:	Not applicable

II. INDICATIONS FOR USE

Ethicon™ OMNEX™ Surgical Sealant is indicated for use in vascular reconstructions to achieve adjunctive hemostasis by mechanically sealing areas of leakage.

III. CONTRAINDICATIONS

- Do not use on patients with known hypersensitivity to cyanoacrylate or formaldehyde.
- Not for intravascular use.

IV. WARNINGS AND PRECAUTIONS

The Warnings and Precautions can be found in the product labeling.

V. DEVICE DESCRIPTION

Ethicon™ OMNEX™ Surgical Sealant (Ethicon™ OMNEX™) is a synthetic tissue sealant consisting of a blend of two monomers, 2-octyl cyanoacrylate (2-OCA) and butyl lactoyl cyanoacylate (BLCA). The liquid formulation is

contained in a crushable glass ampoule, which is housed in a single-use delivery device. The formulation is passed through a porous disc containing an initiator, mixed in a chamber, and delivered through a cannula. Following standard closure techniques using sutures, staples and/or clips, Ethicon™ OMNEX™ is applied to the anastomotic closure line. The polymerizing formulation is spread using the cannula such that it wets and intimately contacts the anastomotic closure. When polymerization is complete, a film is formed that mechanically interlocks the tissue and/or non-biological materials (i.e. synthetic graft sutures, staples, clips) and creates a flexible physical seal, independent of the body's clotting mechanism. The formation of this flexible physical seal prevents leakage of blood along the anastomotic closure line. Ethicon™ OMNEX™ begins to polymerize immediately on mixing with the initiator and forms a physical seal within 2 minutes after application. Ethicon™ OMNEX™ has been formulated to provide a strong physical seal that remains in place beyond the time required for natural healing, and eventually degrades via hydrolytic chain scission (over approximately 36 months), breaking down into smaller absorbable fragments.

The sterile device is provided as a packaged single-use applicator and stored at room temperature.

VI. ALTERNATIVE PRACTICES AND PROCEDURES

There are several other alternatives for the control of bleeding, including use of direct pressure, sutures, staples, and/or electrocautery. In addition, absorbable hemostatic agents such as bovine/porcine gelatin powder and sponges, and hemostats made from collagen as well as oxidized cellulose are commercially available and are used for stopping bleeding. Synthetic liquid polymer sealants, such as those consisting of hydrogel or bovine albumin/gluteraldehyde, are also available to control bleeding in cardiac and vascular surgery. Each alternative has its own advantages and disadvantages.

VII. MARKETING HISTORY

Ethicon™ OMNEX™ Surgical Sealant has regulatory approval for commercial distribution starting in 2006 and has been sold in the following countries/regions: European Union, Russia, Czech Republic, Australia, Brazil, Mexico, Argentina, Chile, Uruguay, Columbia, Venezuela, Peru, South Africa, Israel, and Lebanon. Ethicon™ OMNEX™ Surgical Sealant has not been withdrawn from marketing for any reason relating to the safety or effectiveness of the device.

VIII. POTENTIAL ADVERSE EFFECTS OF THE DEVICE ON HEALTH

Below is a list of the potential adverse effects (e.g., complications) associated with the use of this class of surgical sealants:

- Hypersensitivity reaction such as swelling or edema at the application site
- Application of the sealant to tissue not targeted for the procedure

- Failure of the sealant to adhere to the tissue
- Thrombosis and thromboembolism

Below is a list of the potential adverse effects (e.g., complications) associated with cardiac and vascular procedures:

- Adhesions
- Anastomotic pseudoaneurysm
- Aortic insufficiency
- Cardiac tamponade
- Cerebral emboli
- Coagulopathy
- Death or irreversible morbidity
- Dissection
- Edema
- Erythema
- Hematoma
- Hemorrhage
- Infection
- Injury to normal vessels or tissue
- Ischemia
- Lymphocele/lymph fistula
- Myocardial infarction
- Neurological deficits
- Organ system dysfunction/failure
- Pain
- Paraplegia
- Pleural effusion
- Pulmonary emboli
- Renal dysfunction/failure
- Stroke or cerebral infarction
- Thrombosis
- Vasospasm
- Vessel rupture and hemorrhage

For the specific adverse events that occurred in the clinical studies, please see Section X below.

IX. SUMMARY OF PRECLINICAL STUDIES

A. Laboratory Studies

***In Vitro* Testing**

In vitro (bench) testing was conducted to characterize sterilized Ethicon™ OMNEX™ Surgical Sealant and to ensure that the same product is being manufactured on a lot-to-lot basis. The findings from the *in vitro* testing are

summarized in Table 1. As illustrated in the table, all results support the safety and effectiveness of the product.

Table 1. Summary of *In Vitro* Testing and Results

In Vitro Test:	Test Article:	Results:
Viscosity Determination	Final Formulation: Ethicon™ OMNEX™	Acceptable internal resistance to flow
Average Setting Time	Final Formulation: Ethicon™ OMNEX™	Sufficient polymerization to adequately seal the suture line within an average of < 15 seconds
Working Time	Final Formulation: Ethicon™ OMNEX™	Time available to surgeon to use sealant before it polymerizes within the applicator is > 120 seconds
Identity	Final Formulation: Ethicon™ OMNEX™	All lots tested matched reference identity chromatogram
Molecular Weight and Polydispersity Index Analysis	Final Formulation: Ethicon™ OMNEX™	Molecular weight range and polydispersity index (PDI) demonstrate consistency of product across lots
Delivery System Performance	Final Formulation: Ethicon™ OMNEX™	Product consistently delivers between 220 µl to 235 µl of sealant per device
Degradation Study	Final Formulation: Ethicon™ OMNEX™	In saline at 50°C, complete degradation is estimated to be between 2.1 and 2.5 yrs

Sterilization

Ethicon™ OMNEX™ Surgical Sealant is dry heat sterilized after packaging into glass ampoules, and then terminally sterilized with Ethylene Oxide in compliance with ANSI/AAMI/ISO ST63:2002, ANSI/AAMI/ISO 11135, ANSI/AAMI/ISO 10993-7, and ANSI/AAMI/ISO TIR 19:1998. Based on the design, manufacturing, and the validated sterilization procedure for Ethicon™ OMNEX™ Surgical Sealant, the test results indicate that the device will maintain a Sterility Assurance Level (SAL) of 10^{-6} when sterilized according to the validated procedures.

Packaging and Shelf Life

Ethicon™ OMNEX™ Surgical Sealant is packaged in a delivery system that contains a single ampoule sealing the monomers and stabilizers until ready for use. The product is sealed within a Tyvek® pouch, with 4 pouches per box. Qualification testing was performed for packaging design performance, packaging shelf-life, and device shelf-life for the Ethicon™ OMNEX™ Surgical Sealant. A 12-month shelf-life has been established for the product.

Biocompatibility

Biocompatibility of Ethicon™ OMNEX™ Surgical Sealant was evaluated in accordance with ISO 10993-1, Biological Evaluation of the Medical Devices – Part 1: Evaluation and Testing and FDA Blue Book Memorandum G95-1. All testing was conducted at Toxikon, Inc. in accordance with FDA good laboratory practice (GLP) regulations (21 CFR, Part 58).

Tests conducted on Ethicon™ OMNEX™ Surgical Sealant were appropriate for an implant device that is in permanent contact with tissue (> 30 days). The findings from the biocompatibility testing are summarized in Table 2. All test results indicated that the materials and processes used to manufacture Ethicon™ OMNEX™ Surgical Sealant and delivery system are biocompatible and suitable for their intended use.

Table 2. Summary of Biocompatibility Testing and Results

Biocompatibility Test:	Test Article:	Results:
Cytotoxicity	Final Formulation: Ethicon™ OMNEX™:<br>• Polymerized film in MEM +10% FBS	Non-cytotoxic
Intracutaneous Reactivity	Final Formulation: Ethicon™ OMNEX™:<br>• Unpolymerized sealant* in saline<br>• Polymerized film in sesame oil	Non-irritating
Dermal Sensitization	Final Formulation: Ethicon™ OMNEX™:<br>• Unpolymerized sealant* in saline<br>• Polymerized film in sesame oil	Not a sensitizer
Acute Toxicity	Final Formulation: Ethicon™ OMNEX™:<br>• Unpolymerized sealant* in saline<br>• Polymerized film in sesame oil	Not an acute toxin
Pyrogenicity	Final Formulation: Ethicon™ OMNEX™:<br>• Unpolymerized sealant* in saline	Non-pyrogenic
Hemolysis	Final Formulation: Ethicon™ OMNEX™:<br>• Unpolymerized sealant* in saline	Non-hemolytic
Ames Assay	Final Formulation: Ethicon™ OMNEX™:<br>• Unpolymerized sealant* in saline<br>• Polymerized film in sesame oil	Non-mutagenic in bacteria
Mouse Lymphoma	Final Formulation: Ethicon™ OMNEX™:<br>• Polymerized film in Fisher's +5%HS	Non-mutagenic in mammalian cells
In Vitro Chromosomal	Final Formulation: Ethicon™	Non-clastogenic *in*

P060029: FDA Summary of Safety and Effectiveness Data page 5

9

Biocompatibility Test:	Test Article:	Results:
Aberration	OMNEX™:<br>• Polymerized film in Ham's F12	*vitro*
In Vivo Mouse Micronucleus	Final Formulation: Ethicon™ OMNEX™:<br>• Unpolymerized sealant* in saline<br>• Polymerized film in sesame oil	Non-clastogenic *in vivo*
Cytotoxicity	Final Formulation: Ethicon™ OMNEX™:<br>• Polymerized film in MEM +10%FBS	Non-cytotoxic
Intracutaneous Reactivity	Final Formulation: Ethicon™ OMNEX™:<br>• Unpolymerized sealant* in saline<br>• Polymerized film in sesame oil	Non-irritating

*sealant polymerized during the conduct of the test

Because this product is applied on the outside of the vessel and is not intended for direct contact with blood, FDA generally does not request full hemocompatibility testing. However, reports from plasma recalcification time and complement activation testing were provided for this device, with no adverse findings.

For all tests conducted using non-saline extracts (e.g., tissue culture media, oil), extractions were conducted using fully polymerized product. These extractions do not allow for assessment of the toxicity of starting or intermediate compounds. In addition, none of the biocompatibility screening tests are designed to investigate the toxicity of the final breakdown products. Therefore, results from a variety of animal implant studies (Table 3), as well as results from a literature review, were used to address the toxicity of the starting, intermediate, and final breakdown products of this device. The literature review was based on the amount of chemicals present in 1000 µl of sealant, which is equivalent to four fully expressed units of Ethicon™ OMNEX™ Surgical Sealant (4 x 250 µl). Although stabilizer components of the product have been classified as potential carcinogens, the quantities of these components in extracts of four units of polymerizing or polymerized sealants were below detection levels in a battery of genotoxicity tests.

Results from the animal implant studies, and data from the literature review, were used to justify omission of conventional muscle implant, sub-chronic toxicity, chronic toxicity and carcinogenicity biocompatibility screening studies. Instead, several animal implant studies were conducted, including a 24-month high dose (*in situ* polymerization) implant study in rats. Results from these animal studies are provided in Table 3 below.

B. Animal Studies

Ethicon™ OMNEX™ Surgical Sealant was subjected to a series of acute and chronic animal studies. The intent of the studies was to demonstrate safety of the device by acceptable functional performance of the subject devices in an *in vivo* setting. Additionally, the studies were intended to ensure that the devices do not cause adverse biological responses.

Ethicon, Inc. has conducted five (5) preclinical studies that evaluated the safety of the Ethicon™ OMNEX™ Surgical Sealant. These studies were conducted in accordance with Good Laboratory Practices (GLP) per 21 CFR§ 58. All product was implanted successfully, and all animals survived to the pre-determined study endpoints. Table 3 outlines the animal studies performed and the relevant findings for each study.

Table 3. Summary of Animal Studies Performed

Study Type:	Test Articles:	Number of Animals and Amount of Product Tested:	Follow-Up Duration:	Relevant Findings:
Ovine Graft Study (Iliac anastomosis)	Test Article: Final Formulation - Ethicon™ OMNEX™ Control: Oxidized regenerated cellulose	8 Ethicon™ OMNEX™ and 8 Control sites in 12 animals. Dose: As required to seal anastomosis	Intervals up to 18 month	Moderate to marked chronic active reaction at 2 and 4 weeks, decreasing to a mild reaction at 6 and 18 months. The investigators concluded that use of the surgical sealant is effective and does not result in any adverse complications of vessel patency, vessel stenosis, and degree of tissue reactivity.
Ovine Venotomy Study	Test article: Final Formulation - Ethicon™ OMNEX™. Control: Competitor	16 animals: 8 Ethicon™ OMNEX™ and 16 Controls (2 groups). Dose: As	1 month	Minimal to moderate pyogranulomatous inflammation. Due to the 1 month duration of the study,

P060029: FDA Summary of Safety and Effectiveness Data page 7 11

Study Type:	Test Articles:	Number of Animals and Amount of Product Tested:	Follow-Up Duration:	Relevant Findings:
	products	required to seal anastomosis		resolution of inflammation was not observed, as was found in the longer term studies.
Rodent Sciatic Nerve Study	Test article: Final Formulation - Ethicon™ OMNEX™ Control: saline	20 rats: 10 Ethicon™ OMNEX™ and 10 Controls. Dose: ≈ 10 µl	2 week	Mild tissue reaction. Due to the 2 week duration of the study, resolution of inflammation was not observed, as was found in the longer term studies.
Rodent Intraperitoneal Implantation	Test article: Final Formulation - Ethicon™ OMNEX™	20 rats: 20 Ethicon™ OMNEX™ no controls. Dose: 4 x 10 µl/rat	Intervals up to 23 month	Mild to marked macrophage response. Based on the change in sealant mass and morphological changes observed in this study and under the specified conditions, the estimated mass loss at 23 months is 75% of the initial mass. This predicts at least 90% mass loss in a time range of 30 to 36 months, considering that the degradation profile remains constant.
Rodent Subcutaneous Implantation	Test article: Ethicon™ OMNEX™	560 rats: 280 Ethicon™ OMNEX™	24 month	Minimal to mild chronic granulomatous

P060029: FDA Summary of Safety and Effectiveness Data page 8 12

Study Type:	Test Articles:	Number of Animals and Amount of Product Tested:	Follow-Up Duration:	Relevant Findings:
	Control: saline	and 280 Control studied at various time points to 2 years. Dose: 2 x 100 µl/rat		inflammation/ fibrosis. A low but increased incidence of well-known rodent-specific fibrosarcomas not relevant for humans. Ethicon™ OMNEX™ present throughout study. No adverse local reaction, systemic toxicity, or evidence of carcinogenicity likely to be applicable to the product's use in humans. However, the product, which is designed to be degradable, did not degrade to any significant extent during the 24-month implantation period and as such the long-term safety effects of the degradation products have not been established.

X. SUMMARY OF PRIMARY CLINICAL STUDIES

Three clinical trials were conducted to support the safety and effectiveness of the Ethicon™ OMNEX™ Surgical Sealant. A feasibility study was conducted under IDE # G030143, and enrolled 10 patients at two (2) centers in the United States (US) and evaluated the safety and feasibility of sealing anastomotic suture lines with Ethicon™ OMNEX™ Surgical Sealant to provide hemostasis in patients undergoing arteriovenous shunt procedures receiving an expanded polytetrafluoroethylene (ePTFE) graft. The Multi-Center Pivotal Study enrolled 151 patients at 13 centers in the US and the European Union (EU) and evaluated the safety and effectiveness of Ethicon™ OMNEX™ Surgical Sealant for use as an anastomotic sealant to provide hemostasis in patients undergoing vascular reconstruction procedures receiving an ePTFE graft, as compared to a standard of care control. The Multi-Center Registry Study enrolled 105 patients at 5 centers in Germany, and evaluated the safety and effectiveness of sealing anastomotic suture lines with Ethicon™ OMNEX™ Surgical Sealant in patients undergoing vascular reconstruction procedures using various types of graft materials.

All of the trials used the final formulation of the Ethicon™ OMNEX™ Surgical Sealant. However, the delivery system was slightly different in each of the three trials, with the commercial design of the delivery system being used in the Multi-Center Registry Study. Non-clinical bench, biocompatibility, and animal testing demonstrated that Ethicon™ OMNEX™ Surgical Sealant delivered across various iterations of the delivery system design have comparable characteristics and performance. Although the feasibility and pivotal clinical data were collected on product with a slightly different delivery system design, the data support the approval of the Ethicon™ OMNEX™ Surgical Sealant with the commercial design of the delivery system. Summaries of these clinical trials are presented below.

Feasibility Study

A. Study Design

The study was a prospective, non-randomized, controlled, multi-center trial to evaluate the safety and feasibility of Ethicon™ OMNEX™ Surgical Sealant to seal anastomotic suture lines in patients undergoing arteriovenous (AV) shunt procedures receiving an ePTFE graft for dialysis. Ten (10) patients were enrolled at two (2) centers in the US. The objective of the study was to collect clinical data concerning the safety and feasibility of Ethicon™ OMNEX™ Surgical Sealant as an adjunctive anastomotic sealant to provide hemostasis.

Subjects underwent AV graft placement using standard surgical procedures (according to the Instructions for Use for the graft). After the graft was sutured in place, the vessel was clamped to prevent bleeding through the suture line, the graft and tissue surfaces were blotted dry, and small drops of Ethicon™ OMNEX™ Surgical Sealant were expressed from the delivery system and spread into a thin film along the anastomotic closure line.

B. Safety and Effectiveness Results

The clinical results show that the mean elapsed time from clamp release to observed hemostasis was 9.1 seconds (range 0 – 91 seconds). The percent of patients with immediate hemostasis was 90% (9/10). Immediate hemostasis was defined as zero (0) minutes from the time of clamp release to achieving hemostasis. Time to hemostasis was determined using a calibrated stopwatch provided to each study site for use in the study. The percent of patients achieving hemostasis at 1, 5, and 10 minutes were 90% (9/10), 100% (10/10), and 100% (10/10), respectively. No additional adjunctive measures, although allowed, were necessary to achieve hemostasis.

There was one possible device-related event during the course of the study. This adverse event was an occlusion of the graft and native vessel noted at the 12-week visit, an expected event for vascular access grafts that did not raise concerns for expansion to the pivotal study. No other device-related events were reported for the other nine patients in the study.

Multicenter Pivotal Study

A. Study Design

Patients were enrolled between April 26, 2004 and January 18, 2005.. The database for this PMA reflected data collected through April 2005 and included 151 patients. There were 13 investigational sites, 10 in the United States and 3 in Europe.

The study was a prospective, randomized, controlled, open-label, multi-center trial conducted to evaluate the safety and effectiveness of Ethicon™ OMNEX™ Surgical Sealant (Ethicon™ OMNEX™) versus Control to seal anastomotic suture lines in patients undergoing vascular reconstruction procedures receiving an ePTFE graft. The 151 patients were randomized 2:1, Ethicon™ OMNEX™ versus Control (a commercially available, adjunctive sealant comprised of oxidized regenerated cellulose, a legally marketed alternative with similar indications for use). Randomization was stratified based on the type of procedure, that is, whether the patient was undergoing a femoral bypass or vascular access procedure for dialysis. The objective of the study was to collect clinical data concerning the safety and effectiveness of Ethicon™ OMNEX™ for use as an anastomotic sealant to provide hemostasis and to show superiority to the control, which was considered the standard of care. The patients were evaluated during surgery, before discharge, and at 4 and 12 weeks follow-up.

Frequentist statistics were used to analyze the primary effectiveness endpoint, time to hemostasis from clamp release. For femoral bypass patients with more than one anastomotic site treated, the site with the longest time to hemostasis was used in the analysis. The primary effectiveness analysis was

performed in two stages. The first stage was a test of non-inferiority, and the second a test of superiority, conditioning on the test for non-inferiority being significant. The statistical hypotheses for the first stage were as follows:

$$H_O: \mu_T - \mu_C = \delta$$
$$H_A: \mu_T - \mu_C < \delta,$$

where μ_T and μ_C are the population mean times to hemostasis for the Ethicon™ OMNEX™ and Control groups, respectively. The non-inferiority margin, δ, is 1 minute. Rejection of this null hypothesis ($p < 0.05$), in favor of the alternative, would provide evidence that Ethicon OMNEX is non-inferior to Control in its mean time to hemostasis. If, and only if, this null hypothesis is rejected ($p<0.05$), then the following statistical hypotheses was tested in the second stage:

$$H_O: \mu_T - \mu_C = 0$$
$$H_A: \mu_T - \mu_C \neq 0.$$

Rejection of this null hypothesis ($p < 0.05$) in favor of the Ethicon™ OMNEX™ treatment group would provide evidence of the superiority of Ethicon™ OMNEX™ over the Control in terms of mean time to hemostasis.

A minimum sample size of 100 Ethicon™ OMNEX™ and 50 Controls was necessary to provide 80% power ($p < 0.05$) to reject the non-inferiority null hypothesis. Moreover, this sample size also provided 80% power ($p < 0.05$) to reject the second stage superiority null hypothesis.

A secondary effectiveness analysis was performed to determine the proportion of subjects achieving immediate hemostasis or by 1, 5, or 10 minutes after clamp release. As with the primary effectiveness variable, femoral bypass patients with more than one anastomotic site treated had the site with the longest time to hemostasis used. These data were analyzed by the Cochran-Mantel-Haenszel procedure, stratified by the cross-classification of the study center and procedure. An additional effectiveness analysis was performed to ascertain the frequency of use of additional adjunctive measures to achieve hemostasis. Use of additional adjunctive agents was analyzed by the Cochran-Mantel-Haenszel procedure, stratified by the cross-classification of study center and procedure. Femoral bypass patients who required an additional agent for any anastomotic site were classified as having required use of the additional agent. In addition, safety was assessed by comparing adverse events and device-related adverse events through the 4-week and 12-week follow-up period. These analyses were not powered for sample size.

An independent medical monitor was used in this study to evaluate all safety-related events.

1. Clinical Inclusion and Exclusion Criteria

Enrollment in the Ethicon Omnex study was limited to patients who met the following inclusion criteria:

- Patients undergoing femoral bypass procedures or AV shunt procedures for hemodialysis access using ePTFE vascular grafts
- Prior written informed consent
- Age ≥ 18 years
- Patient agreement to return for follow-up evaluations.

Patients were not permitted to enroll in the Ethicon OMNEX study if they met any of the following exclusion criteria:

- Patients with a known hypersensitivity for formaldehyde or cyanoacrylate
- Women with known pregnancy
- Current or recent (< 6 months) participation in another investigational study of surgical/therapeutic device, drug, or biologic
- Receiving anti-vitamin K anticoagulants within 4 days prior to surgery
- Receiving low molecular weight heparins within 4 days prior to surgery
- For femoral bypass procedures or AV shunt procedures, utilization of a gelatin or collagen coated graft material
- For femoral bypass procedures or AV shunt procedures, utilization of an autologous graft

2. Follow-up Schedule

All patients were scheduled for follow-up examinations at 48 hours, 4 weeks, and 12 weeks postoperatively.

Postoperatively, the objective parameters measured during the study included distal radial pulses (for AV shunt procedures), and both ankle and brachial pressure for determination of ankle-brachial index (for patients who had femoral bypass procedures). All patients also had standard clinical evaluations for adverse event assessments. Adverse events and complications were recorded at all visits.

3. Clinical Endpoints

With regards to safety, endpoints included adverse events and device-related adverse events during the procedure hospitalization and from hospital discharge through the entire 12-week follow-up period.

With regards to effectiveness, the primary endpoint was the elapsed time from surgical clamp release to hemostasis, recorded in seconds. For

femoral bypass patients with more than one anastomotic site treated, the site with the longest time to hemostasis was used in the analysis.

The secondary effectiveness endpoints were:
- Proportions of subjects achieving hemostasis at t = 0 (immediate) or by 1, 5, or 10 minutes of post clamp release
- Frequency of use of additional adjunctive measures to achieve hemostasis [e.g. additional applications of Ethicon™ OMNEX™ (treatment arm only) or other sealants (Control arm only), stitches, pledgets, administration of protamine, or other standard of care)

B. Accountability of PMA Cohort

At the time of database lock, 266 patients were screened with 151 enrolled in the study, 151 (100%) completed treatment, and 126 patients (83%) were available for analysis at the completion of the study, the 12 week post-operative visit.

Table 4. Patient Accountability

	Ethicon™ OMNEX™ % (n)	Control % (n)
**Screened**	266	
**Randomized**	100% (101)	100% (50)
**Treated**	100% (101)	100% (50)
**Completed Surgery**	100% (101)	100% (50)
**Died in Hospital**	2% (2)	0% (0)
**Discharged**	98% (99)	100% (50)
**Completed 4 Week Follow-up**	91% (92)	98% (49)
**Completed Study (12 Week Follow-up)**	80% (81)	90% (45)

C. Study Population Demographics and Baseline Parameters

The demographics of the study population are typical for a peripheral vascular anastomosis study performed in the US. Table 5 depicts the patient demographics.

Table 5. Patient Demographics by Age, Gender, and Surgical Procedure

	Ethicon™ OMNEX™ (n=101)	Control (n=50)
**Age**		
Mean ± SD	60.8 ± 14.3	61.4 ± 13.9
Median	62.0	61.0
Range	21 – 96	29 – 90
**Gender**		
Males: n (%)	66 (65.4%)	28 (56.0%)
Females: n (%)	35 (34.6%)	22 (44.0%)

P060029: FDA Summary of Safety and Effectiveness Data page 14

18

	Ethicon™ OMNEX™ (n=101)	Control (n=50)
**Race**		
Asian: n (%)	0 (0%)	0 (0%)
Black: n (%)	31 (30.7%)	14 (28%)
Hispanic: n (%)	7 (6.9%)	0 (0%)
White: n (%)	62 (61.4%)	36 (72%)
Other: n (%)	1 (1.0%)	0 (0%)
**Procedure**		
Femoral Bypass: n (%)	46 (45.5%)	23 (46.0%)
AV Access for Hemodialysis: n (%)	55 (54.5%)	27 (54.0%)

Gender Analysis:

In this study, women comprised 35% of the Ethicon™ OMNEX™ group versus 44% in the Control group. There was no significant difference in gender distribution between the Ethicon™ OMNEX™ and Control groups (P = 0.26). The gender distributions are consistent with the patient populations who have undergone femoral bypass and arteriovenous access for hemodialysis, compared to data available in the American College of Surgeons – National Surgical Quality Improvement Program (Marcus, RJ, Marcus, DA, Sureshkumar, KK, Hussain, SM, and McGill, RL. Gender differences in vascular access in hemodialysis patients in the United States: Developing strategies for improving access outcome. Gender Medicine, 4(3):193-204, 2007).

Treatment Sites per Patient:

Since Ethicon™ OMNEX™ or the Control material may have been used in more than one location in an individual patient, Table 6 below shows the number of sites treated/patient in this study. However, the effectiveness results were based on per-patient statistics.

Table 6. Treatment Sites per Patient

	Ethicon™ OMNEX™	Control
**Total Number of Patients Treated:**	101	50
Number of Patients with 1 Site Treated:	57	27
Number of Patients with 2 Sites Treated:	40	21
Number of Patients with 3 Sites Treated:	4	2
**Total Number of Sites Treated:**	149	75

D. Safety and Effectiveness Results

1. Safety Results

The analysis of safety was based on the total cohort of 151 patients and the 126 patients that completed the 12 week follow-up period. As a key safety outcome, there were no unanticipated adverse device effects (UADE) in this investigation.

Adverse effects that occurred in the PMA clinical study:
The adverse events in this study are presented below in Tables 7 and 8. Table 7 provides a summary of vascular and bleeding complication adverse events reported for the Ethicon™ OMNEX™ treated and Control treated patients.

Table 7: Vascular or Bleeding Complications Adverse Events

	**Ethicon™ OMNEX™ (n=101) % (n)**	**Control (n=50) % (n)**
**Number of Patients with at Least 1 Vascular or Bleeding Complication**	22.8% (23)	40% (20)
**Bleeding Complications**		
Bleeding, procedure	2% (2)	0% (0)
Bleeding, post procedure	2% (2)	2% (1)
Hematoma	2% (2)	12% (6)
Coagulopathy	1% (1)	0% (0)
**Vascular Complications**		
Occlusion of Graft/Vessel	11.9% (12)	16% (8)
Edema	5% (5)	8% (4)
Thrombosis	5% (5)	6% (3)

Results show that the number of patients with at least one vascular or bleeding complication was greater in the control than Ethicon™ OMNEX™, and the difference was statistically significant, although the study was not powered for this analysis ($p < 0.035$).

In addition, the results are similar between the two treatment groups and are representative of events expected from patients undergoing vascular surgery for vascular access or occlusive vascular disease with the exception of hematoma (control 12% and Ethicon™ OMNEX™ 2%; $p < 0.016$), although the study was not powered for this analysis.

Table 8 shows all other adverse events reported by three or more patients treated with Ethicon™ OMNEX™ or control.

P060029: FDA Summary of Safety and Effectiveness Data page 16 20

Table 8: Other Adverse Event[1] Complications Reported by Three or More Patients Treated

	Ethicon™ OMNEX™ (n=101) % (n)	Control (n=50) % (n)
Infection[2]	9.9% (10)	16% (8)
Pain	8.9% (9)	10% (5)
Erythema	8.9% (9)	2% (1)
Wound Infection[3]	7.9% (8)	4% (2)
Dehiscence	5% (5)	0% (0)
Renal Failure	4% (4)	4% (2)
Lymphocele/Lymph Fistula	3% (3)	0% (0)

[1] All adverse events other than vascular or bleeding complications reported
[2] Infection was defined as non-wound infections reported
[3] Wound infection was defined as surgical incision site infections reported

2. Effectiveness Results

Primary Endpoint
The primary effectiveness outcome parameter measured was time to hemostasis for patients treated with Ethicon™ OMNEX™ to that of patients treated with the Control. For femoral bypass patients with more than one anastomotic site treated, the site with the longest time to hemostasis was used in the analysis. These results are presented in Table 9.

Table 9. Time to Hemostasis (sec)[1] Summary – All Patients

	Ethicon™ OMNEX™ (n=101)	Control (n=50)
Mean Time to Hemostasis [2,3]	119.3	403.8

[1] The anastomotic site with the longest time to hemostasis was used for femoral bypass patients with multiple sites treated. All times > 10 minutes were replaced by 10 minutes.
[2] Adjusted for study center and type of procedure
[3] Test of hypothesis that Ethicon™ OMNEX™ mean is no more than one minute longer than that of the control; test of non-inferiority p-value is < 0.001; Test of superiority p-value is < 0.001.

Multiple analyses were conducted to evaluate the effectiveness data by procedural type and by patient. These analyses demonstrated that all study objectives were met. The results of the study showed that patients who received Ethicon™ OMNEX™ had a statistically significant faster time to hemostasis than that of the control ($p < 0.001$).

Secondary Endpoints

The secondary effectiveness endpoints were:

- Number of patients who achieved hemostasis within 0 (immediate), 1, 5, and 10 minutes
- Number of patients who required additional adjunctive agents in order to achieve hemostasis

Table 10. Time to Hemostasis[1] by Minute Intervals for All Procedures - All Patients

Interval	Ethicon™ OMNEX™ (n=101) % (n)	Control (n=50) % (n)
0 (immediate)	54.5% (55)	10% (5)
0 - 1 minute	60.4% (61)	14% (7)
0 - 5 minutes	88.1% (89)	32% (16)
0 - 10 minutes	93.1% (94)	58% (29)
> 10 minutes	6.9% (7)	42% (21)

[1] The anastomotic site with the longest time to hemostasis was used for femoral bypass patients with multiple sites treated. All times > 10 minutes were replaced by 10 minutes

As with the primary variable, femoral bypass patients with more than one anastomotic site treated had the site with the longest time to hemostasis used. These data were analyzed by the Cochran-Mantel-Haenszel procedure, stratified by the cross-classification of the study center and procedure.

Table 11. Use of Additional Adjunctive Agents to Achieve Hemostasis – All Patients

	Ethicon™ OMNEX™ (n=101) % (n)	Control (n=50) % (n)
At least one additional agent required[1]	30.7% (31)	44 % (22)
One additional unit of assigned treatment	10.9% (11)	30% (15)
Stitches	6.9% (7)	14% (7)
Pledgets	0% (0)	0% (0)
Protamine	6.9% (7)	16% (8)
Other	11.9% (12)	6% (3)

[1] Total number of individual agents may exceed the number of patients who had at least one agent used because patients may have had multiple agents used and because multiple anastomotic sites were treated in femoral bypass patients

Use of additional adjunctive agents was analyzed by the Cochran-Mantel-Haenszel procedure, stratified by the cross-classification of study center and procedure. Femoral bypass patients who required an additional agent for any anastomotic site were classified as having required use of the

additional agent. A greater proportion of patients in the control group (44.0%) required at least one additional agent than patients in the Ethicon OMNEX group (31%), although the difference did not achieve statistical significance (p = 0.08).

During the clinical investigation, the number of Ethicon™ OMNEX™ units used per patient to effectively seal a typical vessel was an average of 1.7 ± 0.88 units (range 1 - 4 units), and for Control, using oxidized regenerated cellulose, 2.2 ± 1.04 units (range 1 - 4 units). The number of Ethicon™ OMNEX™ units used per anastomosis to effectively seal a typical vessel was an average of 1.1 ± 0.25 units (range 1 - 2 units), and for Control, 1.5 ± 0.48 units (range 1 - 2) and is detailed in Table 12 below.

Table 12. Amount of Sealant used in the Clinical Pivotal Study per Patient and by Procedure

	Ethicon™ OMNEX™		Control	
	Patient	Procedure	Patient	Procedure
Mean Units Used	1.7	1.1	2.2	1.5
Std Dev	0.88	0.25	1.04	0.48
Range	1 – 4	1 - 2	1 - 4	0.5 - 2

3. Subgroup Analysis

Inclusion and exclusion criteria were chosen to avoid gender bias. The results of the Pivotal trial demonstrated that there were no significant differences in the Primary Objective (average time to hemostasis) due to gender, with mean results of 107 sec for females and 126 sec for males. In the Control group, the average time to hemostasis was 389 sec for females and 415 sec for males. There were no significant differences in the occurrence of adverse events between males (67%) and females (71%) in the Ethicon™ OMNEX™ treatment group with none in either group directly attributable to Ethicon™ OMNEX™. In the Control group, adverse event occurrence was 71% in males and 68% in females. No important differences in success rate or adverse event rate were detected between males and females in this patient population, and the results presented are representative of both genders.

European Multi-Center Registry Study

A. Study Design

A prospective, non-randomized, single-arm, multi-center trial was conducted in Germany to evaluate the safety and effectiveness of Ethicon™ OMNEX™

to seal anastomotic suture lines in patients undergoing multiple types of vascular reconstruction procedures using various types of graft materials. One hundred five patients (105) were enrolled at five (5) study centers.

B. Safety and Effectiveness Results

1. Safety Results

   There were no significant adverse events related to the product use reported in the Single Arm European Study. The events reported were typical of patients with clinical conditions related to vascular surgeries without the use of Ethicon™ OMNEX™.

2. Effectiveness Results

   The primary effectiveness endpoint was time to hemostasis. Overall, immediate hemostasis (at time 0) was achieved in 71% of the 158 application sites from 105 treated patients. Hemostasis was achieved in 94% of application sites within one minute; in the remaining 6% of application sites, hemostasis was achieved within eight minutes. Overall, mean time to hemostasis by anastomotic site was 23.2 seconds with a 95% confidence interval of 11.0 to 35.3 seconds.

Table 13. Time to Hemostasis

		**AV Access Procedure**	**Bypass & Abdominal Aortic Aneurysm (AAA)**	**Endar-terectomy & Patch**	**Total**
By Patient	# of Patients	7	75	23	105
	Mean (sec)	4.6 ± 8.5	39.6 ± 104.6	23.1 ± 55.9	33.7 ± 92.5
	Median (sec)	0.0	0.0	5.0	0.0
	Range (sec)	0 – 22	0 – 480	0 – 266	0 - 480
By Anastomoses	# of Anastomotic Sites	10	124[1]	24	158
	Mean (sec)	3.2 ± 7.3	25.0 ± 83.7	22.1 ± 54.9	23.2 ± 77.2
	Median (sec)	0.0	0.0	2.5	0.0
	Range (sec)	0 – 22	0 – 480	0 – 266	0 - 480

[1] Time to hemostasis was not captured at one anastomotic site.

Secondary effectiveness variables included the number of anastomotic sites receiving each type of graft material and the number of patients who achieved hemostasis within 0 (immediate), 1, 5, and 10 minutes.

Table 14. Graft Material Used

Graft Material	AV Access Procedure % (n)	Bypass & AAA % (n)	Endarterectomy & Patch % (n)	Total[1] % (n)
PTFE	20% (2)	41.1% (51)	4.2% (1)	34.2% (54)
Dacron	0% (0)	25% (31)	75% (18)	31% (49)
Autologous	70% (7)	33.9% (42)	16.7% (4)	33.5% (53)
Other	10% (1)	0% (0)	4.2% (1)	1.3% (2)

[1] Number of anastomotic sites treated

Table 15. Time to Hemostasis by Graft Type

Graft Type	# of Anastomotic Sites	Time Interval			
		0 (immediate)	0 –1 minute	0-5 minutes	0-10 minutes
PTFE	54	64.8% (35)	85.2% (46)	90.7% (49)	100% (54)
Dacron[1]	48	66.7% (32)	97.9% (47)	100% (48)	100% (48)
Autologous	53	81.1% (43)	98.1% (52)	100% (53)	100% (53)
Other	2	100% (2)	100% (2)	100% (2)	100% (2)

[1] Time to hemostasis was not captured at one anastomotic site in this group

During the clinical investigation, the number of Ethicon™ OMNEX™ units used per patient to effectively seal a typical vessel was an average of 1.56 ± 0.62 units (range 1 - 4 units). For femoral bypass and open AAA repair procedures, the number of Ethicon OMNEX units used per anastomosis to effectively seal a typical vessel was an average of 1.02 ± 0.15 units (range 1 - 2 units). For endarterectomy and patch procedures, the number of Ethicon™ OMNEX™ units used per anastomosis to effectively seal a typical vessel was an average of 1.09 ± 0.29 units (range 1-2 units). For AV access procedures, the number of Ethicon™ OMNEX™ units used per anastomosis to effectively seal a typical vessel was an average of 1.43 ± 0.53 units (range 1 - 2 units), as depicted in Table 16 below.

Table 16. Amount of Sealant used in the European Multi-Center Registry Study per Patient and by Procedure

	Ethicon™ OMNEX™	
	Patient	Procedure
Mean Units Used	1.56	1.02
Std Dev	0.62	.15
Range	1 - 4	1 - 2

P060029: FDA Summary of Safety and Effectiveness Data page 21 25

3. Subgroup Analysis

In the European Registry Study where there was no control group (single-arm), women comprised 24% of the study when all the procedures in the study were combined. No statistical analysis was performed to determine if this ratio is consistent with the general patient population undergoing the same procedures.

XI. PANEL MEETING RECOMMENDATION AND FDA'S POST-PANEL ACTION

In accordance with the provisions of section 515(c)(2) of the act as amended by the Safe Medical Devices Act of 1990, this PMA was not referred to the Circulatory System Devices Panel, an FDA advisory committee, for review and recommendation because the information in the PMA substantially duplicates information previously reviewed by this panel.

XII. CONCLUSION.S DRAWN FROM PRECLINICAL AND CLINICAL STUDIES

A. Safety and Effectiveness Conclusions

The non-clinical studies indicate that the Ethicon™ OMNEX™ Surgical Sealant meets or exceeds safety and performance specifications. Multi-center clinical trials have demonstrated that Ethicon™ OMNEX™ is safe and effective for its intended use as a treatment in vascular reconstructions to achieve adjunctive hemostasis by mechanically sealing areas of leakage. These clinical trials investigated use with various graft types (e.g. ePTFE, Dacron, autologous graft) and in various types of procedures (e.g. femoral bypass, arteriovenous access, AAA repair, endarterectomy) which did not impact the safety or effectiveness of the product. All of the trials used the final formulation of the Ethicon™ OMNEX™ Surgical Sealant. However, the delivery system was slightly different in each of the three trials, with the commercial design of the delivery system being used in the Multi-Center Registry Study. Pre-clinical bench, biocompatibility, and animal testing demonstrated that Ethicon™ OMNEX™ Surgical Sealant delivered across various iterations of the delivery system design have comparable characteristics and performance. Although the feasibility and pivotal clinical data were collected on product with a slightly different delivery system design, the data support the approval of the Ethicon™ OMNEX™ Surgical Sealant with the commercial design of the delivery system. Results from non-clinical and clinical evaluations provide valid scientific evidence and reasonable assurance that the device is safe and effective. Therefore, it is reasonable to conclude that the benefits of use of the device for the target population outweigh the risk of illness or injury when used as indicated in accordance with the labeling and Instructions for Use (IFU).

XIII. CDRH DECISION

CDRH issued an approval order on June 3, 2010. The final conditions of approval cited in the approval order are described below.

The applicant's manufacturing facilities were inspected and found to be in compliance with the device Quality System (QS) regulation (21 CFR 820).

XIV. APPROVAL SPECIFICATIONS

Directions for use: See device labeling.

Hazards to Health from Use of the Device: See Indications, Contraindications, Warnings, Precautions, and Adverse Events in the device labeling.

Post-approval Requirements and Restrictions: See approval order.

Revised: jlg 5/11/2010
Revised: jlg 5/13/2010 (Paul & Ken on AE info and gender citation)
Revised: kjc 5/13/2010
Revised: jlg 5/25/2010

APPENDIX III: SAE REPORTING FORM OF THE BFARM

Fill Test Data #1 — Speichern / Save

Formblatt für die Meldung von schwerwiegenden unerwünschten Ereignissen (SAE) im Rahmen einer klinischen Prüfung oder Leistungsbewertungsprüfung durch Sponsoren und Prüfer nach § 3 Abs. 5 der MPSV

Report form for reporting of serious adverse events (SAE) in clinical trials or performance evaluations for use by sponsors and investigators according to § 3 (5) of the Ordinance on Medical Devices Vigilance

Zuständige Behörde / National Competent Authority:

- ○ BfArM, Bonn
- ○ PEI, Langen
- ○ Andere / Other: ______

Titel der klinischen Prüfung / Title of clinical investigation: ______

Kennzeichen der klinischen Prüfung / Clinical investigation identifier: ______

Meldungsnummer Prüfarzt/Prüfzentrum / Reference number assigned by the investigator: ______

Meldungsnummer Sponsor / Reference number assigned by the sponsor: ______

Datum dieser Meldung / Date of this report: ______

BfArM-Referenz# (falls bekannt) / NCA's case # (if known) ______

Welchen anderen zuständigen Behörden wurde dieser Vorfall ebenfalls gemeldet (europäisch und international)? / Identify to which other NCA's this report was also submitted ______

Art der Meldung / Type of report

- ○ Erstmeldung Prüfer, Prüfstelle / Initial report by investigator, investigation site
- ○ Erstmeldung Sponsor / Initial report by the sponsor
- ○ Follow-up Sponsor / Follow-up report by the sponsor
- ○ Abschlussmeldung Sponsor / Final report by the sponsor
- ○ Kombinierter Erst- und Abschlußbericht Sponsor / Combined initial and final report by the sponsor

Datum des nächsten Berichts (falls zutreffend)/ Expected date of next report (if applicable): ______

Meldung erstattet durch / Report submitted by:

Name: ______

Kontaktperson / Contact person: ______

Straße, Haus-Nr. / Street, house number: ______

Postleitzahl / Postal code: ______ **Ort / City:** ______

Page 1 of 5

**Land / Country:** DE - Deutschland/Germany	**Telefon / Phone:**
**Fax:**	**E-Mail:**

**Prüfarzt / Investigator:**	
**Name:**	
**Kontaktperson / Contact person:**	
**Straße, Haus-Nr. / Street, house number:**	
**Postleitzahl / Postal code:**	**Ort / City:**
**Land / Country:** DE - Deutschland/Germany	**Telefon / Phone:**
**Fax:**	**E-Mail:**

**Angaben zum Medizinprodukt / Medical device information:**
**Name des Produkts / Device name:**
**Modellnummer (falls zutreffend) / Model number (if applicable):**
**Datum der Anwendung des Prüfprodukts / Date of the procedure / first use**
**Explantationsdatum (Nur bei Implantaten) / Explant date (For implants only)**
**Implantationsdauer (Nur bei Implantaten und nur, wenn exakte Implantations- und Explantationsdaten unbekannt) / Duration of Implantation (For implants only. To be filled if the exact implant and explant dates are unknown)**
**Zubehör und/oder mit dem Produkt verbundene Geräte (falls zutreffend) / Accessories / associated devices (if applicable):**
**Eindeutige Produktidentifikation in der klinischen Prüfung / Product identifier in the clinical investigation:**

**Betroffene Person(en) / Person(s) affected:**	
☐ Proband / Subject	
☐ Anwender / User	
☐ Andere Person / Other person	
**Geschlecht / Gender *** unknown - unbekannt	**Geburtsjahr / Year of birth: ***
**Gewicht / Weight [kg]: ***	**Probanden-ID laut Prüfplan / Subject ID according to investigation protocol:**

** Angaben zum Geschlecht, Geburtsjahr und Gewicht nur, falls dies für die Bewertung wesentlich ist oder sein kann. Information on gender, year of birth and weight to be provided if necessary for the assessment of the SAE*

Page 2 of 5

**Schwerwiegendes unerwünschtes Ereignis / Serious adverse event:**
**Datum der Studienprozedur / Date of the study procedure:**
**Datum des Ereignisses / Date of event:**
**Datum, an welchem der Prüfarzt / Prüfeinrichtung informiert wurde / Doctor / Investigation site awareness date:**
**Eintreffen der Meldung beim Sponsor / Sponsor's awareness date:**
**Folgen (eingetreten oder hätten eintreten können) / Outcome (actual or possible):**
☐ Tod / Death
☐ Lebensbedrohende Erkrankung oder Schädigung / Life-threatening illness or injury
☐ Dauernde Beeinträchtigung einer Körperstruktur oder -funktion / Permanent impairment of body structure or a body function
☐ Krankenhausaufnahme oder Verlängerung eines bestehenden Krankenhausaufenthaltes / In-patient hospitalization or prolongation of existing hospitalization
☐ Medizinischer oder chirurgischer Eingriff zur Verhinderung einer dauernden Beeinträchtigung einer Körperstrukur oder –funktion / Medical or surgical intervention to prevent life threatening illness or injury or permanent impairment to a body structure or body function
☐ Schädigung eines Fetus, Fetaltod, kongenitale Fehlbildung oder Geburtsschaden / Foetal distress, foetal death or a congenital abnormality or birth defect
**Ausführliche Beschreibung des Ereignisses / detailed description of the event**
**Maßnahmen der Prüfstelle für die Behandlung des Probanden / Remedial action taken by the investigation site relevant to the care of the subject:**
**NUR VOM SPONSOR ANZUGEBEN: Es handelt sich um / TO BE PROVIDED BY THE SPONSOR ONLY: The event is an (erwartet = in der Risikoanalyse des Sponsors oder im Handbuch des Prüfers betrachtet / expected = addressed in the risk analysis of the sponsor or mentioned in the investigators brochure)**
◯ erwartetes Ereignis / expected event ◯ unerwartetes Ereignis / unexpected event
**Begründung der Einstufung als erwartet oder unerwartet / rationale for the classification as expected or unexpected**

Page 3 of 5

Erste Maßnahmen des Sponsors / Initial actions by the sponsor:

Bezug zur Prüfung / Relation to investigation: (Bitte bei Sponsor- und Prüfzentrenmeldung angeben / to be provided by the sponsor and the investigation site)	ja / yes	möglich / possible	nein / no
Medizinische Prozedur / Medical procedure			
Medizinprodukt / Medical device			
Sonstiges / Other			

Begründung des Bezuges: Bei Bewertungsänderung im Vergleich zur Erst- oder Folgemeldung bitte Begründung angeben / Rationale for the relation; In case of changes compared to initial or follow-up reports of the relation please provide a rationale

Aktuelle Anzahl der in die Prüfung aufgenommenen Patienten / Number of patients currently enrolled:

In der Prüfstelle / In the investigation site:	In allen Prüfstellen (anzugeben vom Sponsor) / All investigations sites (to be specified by the sponsor):

Abschlußmeldung durch den Sponsor / Final report by the sponsor

Ursache des SAE ist: / root cause of the SAE is:

- Medizinische Prozedur / Medical procedure
- Medizinprodukt / Medical device
- Sonstiges / Other

Page 4 of 5

**Untersuchungsergebnisse und Begründung der o.g. Ursacheneinstufung einschließlich einer abschließenden Nutzen-Risikobewertung der klinischen Prüfung unter Berücksichtigung der bisher aufgetretenen SAEs / Investigation results and rationale for the above choosen root cause classification including the final risk-benefit assessment incorporating all SAEs**
**Geplante oder durchgeführte korrektive Maßnahmen mit Zeitplan / Corrective action taken or planned including time schedule:**

Die Vorlage dieser Meldung stellt an sich keine Schlussfolgerung des Herstellers und / oder seines Bevollmächtigten bzw. der zuständigen Behörde dar, dass der Inhalt dieser Meldung vollständig oder zutreffend ist, dass das (die) aufgeführte(n) Medizinprodukt(e) in irgendeiner Weise versagt und/oder zum angegebenen Tod bzw. zur angegebenen Verschlechterung des Gesundheitszustandes einer Person geführt oder beigetragen hat (haben).

Submission of this report does not, in itself, represent a conclusion by the manufacturer and/or authorised representative or the National Competent Authority that the content of this report is complete or accurate, that the medical device(s) listed failed in any manner and/or that the medical device(s) caused or contributed to the alleged death or deterioration in the state of the health of any person.

optionale elektronische Signatur / voluntary electronic signature

Prüfen / Check

Senden / Send

Hinweis: Bitte drucken Sie dieses Formular NICHT aus und senden diesen Ausdruck NICHT an das BfArM. Bitte übermitteln Sie die Daten durch Drücken der Schaltfläche 'Senden'. Sie können an die E-Mail eine Kopie des ausgefüllten Formulars anhängen.

Note: Please do not print this form but send it electronically by clicking the button 'Send' and attach a copy of the form an this email.

Page 5 of 5
